# An amide-based second coordination sphere promotes the dimer pathway of Mn-catalyzed CO_2_-to-CO reduction at low overpotential†

**DOI:** 10.1039/d0sc05679k

**Published:** 2021-02-11

**Authors:** Yong Yang, Mehmed Z. Ertem, Lele Duan

**Affiliations:** Department of Chemistry, Shenzhen Grubbs Institute and Guangdong Provincial Key Laboratory of Energy Materials for Electric Power, Southern University of Science and Technology Shenzhen 518055 China duanll@sustech.edu.cn; Chemistry Division, Energy & Photon Sciences, Brookhaven National Laboratory Upton NY 11973-5000 USA mzertem@bnl.gov

## Abstract

The [*fac*-Mn(bpy)(CO)_3_Br] complex is capable of catalyzing the electrochemical reduction of CO_2_ to CO with high selectivity, moderate activity and large overpotential. Several attempts have been made to lower the overpotential and to enhance the catalytic activity of this complex by manipulating the second-coordination sphere of manganese and using relatively stronger acids to promote the *protonation-first* pathway. We report herein that the complex [*fac*-Mn(bpy-CONHMe)(CO)_3_(MeCN)]^+^ ([**1-MeCN**]^+^; bpy-CONHMe = *N*-methyl-(2,2′-bipyridine)-6-carboxamide) as a pre-catalyst could catalyze the electrochemical reduction of CO_2_ to CO with low overpotential and high activity and selectivity. Combined experimental and computational studies reveal that the amide NH group not only decreases the overpotential of the Mn catalyst by promoting the *dimer* and *protonation-first* pathways in the presence of H_2_O but also enhances the CO_2_ electroreduction activity by facilitating C–OH bond cleavage, making [**1-MeCN**]^+^ an efficient CO_2_ reduction pre-catalyst at low overpotential.

## Abstract 2

The amide NH group decreases the overpotential of Mn-based CO_2_ reduction catalysts by promoting the dimer and protonation-first pathways in the presence of H_2_O and enhances the CO_2_ electroreduction activity by facilitating C–OH bond cleavage.

## Graphical abstract

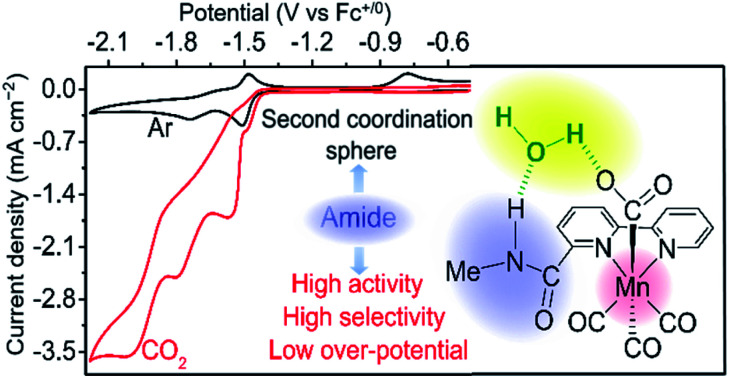

## Introduction

The conversion of carbon dioxide (CO_2_) to value-added fuels and commodity chemicals will contribute to reducing the current global warming effects and solving the energy-related issues of our society.^1,2^ The development of new cost-efficient molecular catalysts that can drive energetically uphill chemical transformations through the electrocatalytic reduction of CO_2_ is an important component of the conversion process.^3,4^ Over the last few decades, significant effort has been devoted to developing transition-metal-based CO_2_ reduction catalysts, such as Mn,^5–13^ Re,^14–20^ Fe,^21–25^ Co,^26–31^ Ni^32–34^ and Ru^35–37^ complexes. Among these catalysts, group VII catalysts, [*fac*-M(N^N)(CO)_3_X] (M = Mn and Re; N^N = diimine ligand; X = monodentate ligand) showed high activity and selectivity for CO_2_ to CO conversion and therefore attracted significant attention.

**Scheme 1:**
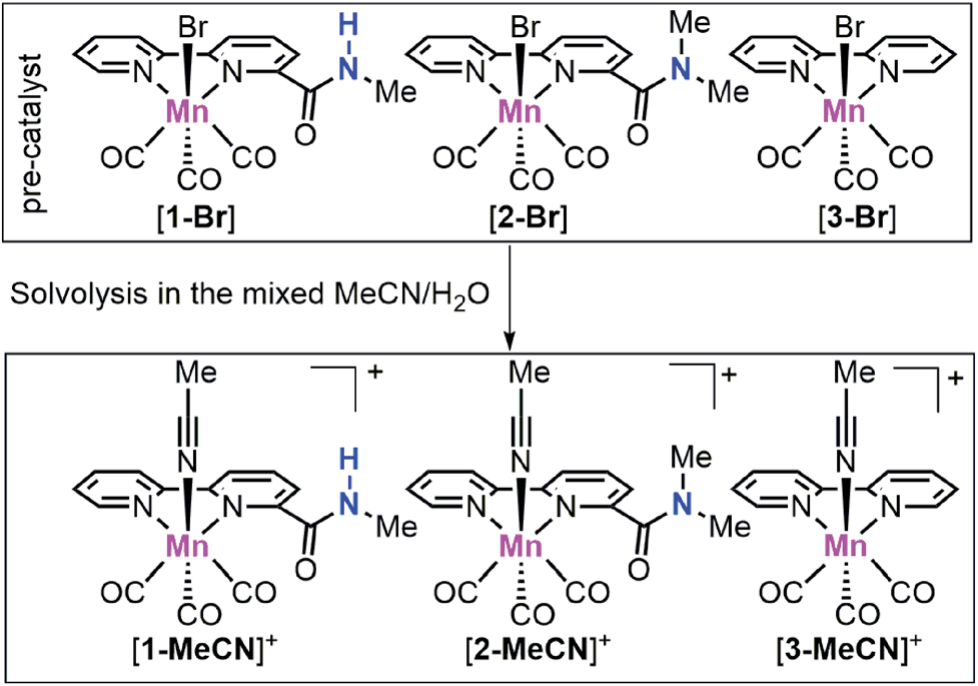
Chemical structures of the manganese complexes [**1-Br**], [**2-Br**] and [**3-Br**] as well as their solvolysis products [**1-MeCN**]^+^, [**2-MeCN**]^+^ and [**3-MeCN**]^+^ under mixed MeCN/H_2_O.

In 2012, Savéant, Costentin and their co-workers reported an Fe porphyrin catalyst with phenolic groups which as a local proton source can enhance the performance of CO_2_ electroreduction,^21^ demonstrating the strong effect of the second coordination sphere on CO_2_ reduction. After that, several research groups have used this strategy to improve CO_2_ reduction activity and designed a few family of Mn/Re based CO_2_ reduction catalysts with functional groups, such as thiourea,^18^ imidazolium,^11,17^ phenol,^38–40^ and ether^41^ moieties as either a hydrogen bond donor or acceptor, resulting in enhanced CO_2_ reduction performances. On the other hand, for earth-abundant Mn-based CO_2_ reduction catalysts, it is difficult to achieve high catalytic activity at low onset potential towards CO_2_ reduction without using strong acids.^6,9,41,42^ Very recently, Nippe, Panetier and their co-workers reported a family of imidazolium-functionalized *fac*-Mn(CO)_3_ bipyridine catalysts, which showed moderate catalytic activity at mild potentials (*ca.* 1.1 mA cm^−2^ at −1.55 V *vs.* Fc^+/0^; *all reduction potentials are reported vs. Fc*^*+/0*^) using H_2_O as the proton source.^11^ For Mn-based CO_2_ reduction catalysts, there are three proposed catalytic pathways for CO production: (i) the *reduction-first* pathway, (ii) the *protonation-first* pathway and (iii) the *dimer* pathway. The *reduction-first* pathway often occurs when weak acids are used as the proton source, while the *protonation-first* pathway generally requires the introduction of stronger acids.^12^ The dimer pathway has been studied by Chardon-Noblat^43^ and Cowan^44^ groups. Their studies indicate that the *fac*-Mn(CO)_3_ bipyridine catalyst could catalyse the CO_2_-to-CO conversion *via* the dimer pathway at a low onset potential but with low catalytic activity. Among these three reaction pathways, the dimer pathway so far has showed the lowest onset potential but on the other hand the catalyst design that could promote this pathway is mostly unexplored. Herein, we tailored the bpy ligand by installing amide groups and prepared [*fac*-Mn(bpy-CONHMe)(CO)_3_Br] ([**1-Br**]; bpy-CONHMe = *N*-methyl-(2,2′-bipyridine)-6-carboxamide; Scheme 1) with an amide –NHMe group with hydrogen bonding and proton donor capability in the second coordination sphere and [*fac*-Mn(bpy-CONMe_2_)(CO)_3_Br] ([**2-Br**]; bpy-CONMe_2_ = *N*,*N*′-dimethyl-(2,2′-bipyridine)-6-carboxamide; Scheme 1), which lacks the amide proton. Under catalytic conditions, complexes [**1-Br**] and [**2-Br**] are respectively converted to [**1-MeCN**]^+^ and [**2-MeCN**]^+^. The amide –NHMe group of [**1-MeCN**]^+^ not only decreases the overpotential requirement of the Mn catalyst by promoting the *dimer* and *protonation-first* pathways in the presence of H_2_O but also enhances the CO_2_ electroreduction activity by facilitating the rate-limiting C–OH bond cleavage. To our knowledge, this is the most active Mn-bpy catalyst utilizing the *dimer pathway* at mild potentials (as positive as −1.50 V) with H_2_O as the proton source.

## Results and discussion

### Synthesis and characterization

Complexes [**1-Br**], [**2-Br**] and [*fac*-Mn(bpy)(CO)_3_Br] ([**3-Br**]) (Scheme 1) were prepared by the reaction between the corresponding ligands and [Mn(CO)_5_Br] according to previously reported procedures (see Fig. S1 and S2 in the ESI†)^7^ and were fully characterized by ^1^H NMR, HR-MS, FTIR and elemental analysis (Fig. S3–S13†). Complexes [*fac*-Mn(bpy-CONHMe)(CO)_3_(MeCN)](OTf) ([**1-MeCN**](OTf)), [*fac*-Mn(bpy-CONMe_2_)(CO)_3_(MeCN)](OTf) ([**2-MeCN**](OTf)) and [*fac*-Mn(bpy)(CO)_3_(MeCN)](OTf) ([**3-MeCN**](OTf)) were synthesized by the reaction between the corresponding ligands and [Mn(CO)_5_(MeCN)](OTf) according to a previously reported procedure^41,45^ and characterized by ^1^H NMR and FTIR (Fig. S11–S18†).

**Fig. 1:**
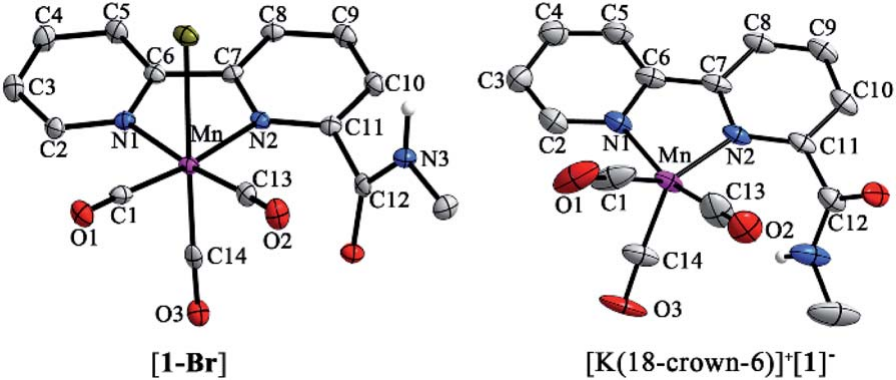
X-ray crystal structures of [**1-Br**] (left) with ellipsoids at the 50% probability level; [K(18-crown-6)]^+^[**1**]^−^ (right) with [K(18-crown-6)]^+^ omitted and ellipsoids at the 35% probability level. Selected bond lengths are the following: complex [**1-Br**] N1–C2 1.345, C2–C3 1.382, C3–C4 1.380, C4–C5 1.388, C5–C6 1.391, C6–C7 1.480, C7–C8 1.385, C8–C9 1.381, C9–C10 1.389, C10–C11 1.383, and C11–N2 1.351; [K(18-crown-6)]^+^[**1**]^−^ N1–C2 1.37, C2–C3 1.363, C3–C4 1.426, C4–C5 1.340, C5–C6 1.406, C6–C7 1.401, C7–C8 1.417, C8–C9 1.354, C9–C10 1.395, C10–C11 1.372, and C11–N2 1.391.

As shown in Fig. 1 (left), the X-ray crystal structure of the complex [**1-Br**] reveals that it crystallized in the space group *P*2(1)/*c* and the geometry is facial octahedral. The second coordination sphere of the amide –NHMe group is out of the bpy plane, while being close to the active site (the Br position), with a dihedral angle N3–C12–C11–N2 of 119.90°. The doubly reduced species [Mn^0^(bpy-CONHMe)˙^−^(CO)_3_] ([**1**]^−^) was chemically generated by the reaction of [**1-Br**] and KC_8_. The single crystals of [K(18-crown-6)]^+^[**1**]^−^ were successfully grown through diffusing pentane into the THF solution of the complex and its X-ray crystal structure is depicted in Fig. 1(right). Compared with [**1-Br**], [**1**]^−^ lost the axial bromide, forming a five-coordinate species that crystallized in the space group *P*2(1)/*c* and its geometry is intermediate between square pyramidal and trigonal bipyramidal with *τ*_5_ = 0.24. For perfect square pyramidal and trigonal bipyramidal geometries, the *τ*_5_ is 0 and 1, respectively.^46^ The dihedral angle N3–C12–C11–N2 of [K(18-crown-6)]^+^[**1**]^−^ is 52.15°, 67.75° smaller than that of [**1-Br**], indicating that the amide –NHMe group is closer to the metal center in the reduced state of the Mn complex in the solid state. The C6–C7 bond in the bpy ring shortens from 1.480 in the crystal structure of [**1-Br**] to 1.401 in [**1**]^−^ (Fig. 1), in good agreement with Kubiak's observations and reflecting the non-innocent character of the bpy ligand upon reduction.^7^ The crystals of complex [**2-Br**] were prepared by employing the same method of [**1-Br**] and crystallized in the space group *P*2(1)/*n* (Fig. S19†). The selected bond lengths and crystallographic data of [**1-Br**], [**2-Br**] and [K(18-crown-6)]^+^[**1**]^−^ are given in Tables S1–S6.†

### Electrochemistry

To demonstrate how the local amide –NHMe group in the secondary coordination sphere influences the electrochemical properties of [*fac*-Mn(bpy-R)(CO)_3_Br] systems, cyclic voltammetry (CV) curves of complexes [**1-Br**], [**2-Br**] and [**3-Br**] were recorded (Fig. S20–S25†). It is well known that the axial bromo ligand of [*fac*-Mn(N^N)(CO)_3_Br] can be partially replaced by CH_3_CN in the acetonitrile solution.^5^ The solvolysis of [**1-Br**] to [**1-MeCN**]Br occurs much faster in mixed acetonitrile/water than in dry CH_3_CN (see the section of ligand exchange in the ESI†). In addition, FTIR measurements in the mixed acetonitrile/water solutions also showed that the solvolysis of the complex [**1-Br**] occurs faster than that of complexes [**2-Br**] and [**3-Br**] (Fig. S26†–S28). Therefore, [**1-MeCN**]^+^ and [**2-MeCN**]^+^ become the dominant species in mixed MeCN/H_2_O (5.5 M H_2_O). *Note: in the following experiments where mixed acetonitrile/water is used as a solvent, the acetonitrile-bound species are regarded as the major species in the solutions although [***1-Br***] and [***2-Br***] are used in the tests; in addition, both the bromo- and acetonitrile-bound complexes are pre-catalysts and the real catalyst form is the reduced, five-coordinate species.* Under the mixed MeCN/H_2_O (5.5 M H_2_O) conditions, the first one-electron reduction of [**1-MeCN**]^+^ occurs at −1.51 V, and the resulting Mn^0^ species undergoes fast acetonitrile dissociation (EC mechanism), forming 5-coordinate [Mn^I^(bpy-CONHMe)˙^−^(CO)_3_] ([**1**]^0^). This [**1**]^0^ monomer is prone to dimerization to yield [**12**]^0^, which could be further reduced at *E* = −1.76 V, leading to the formation of [Mn^0^(bpy-CONHMe)˙^−^(CO)_3_] ([**1**]^−^) (CEC mechanism; Fig. S20†).^7,10,12,41^ In the reverse scan, the oxidation of [**1**]^0^ to [**1**]^+^ was observed at −1.46 V, while the oxidation wave at −0.76 V was assigned to the oxidation of the [**12**]^0^ dimer (Table S7†).^5,6^ Due to the steric influence of the amide –NMe_2_ group, the complex [**2-MeCN**]^+^ exhibited a two-electron reduction wave (Fig. S21a and S23†). The complex [**3-MeCN**]^+^ displayed two reduction waves (Fig. S22a†), and the first and second reduction potentials are more negative than those of [**1-MeCN**]^+^ by −90 and −120 mV, respectively.

**Fig. 2:**
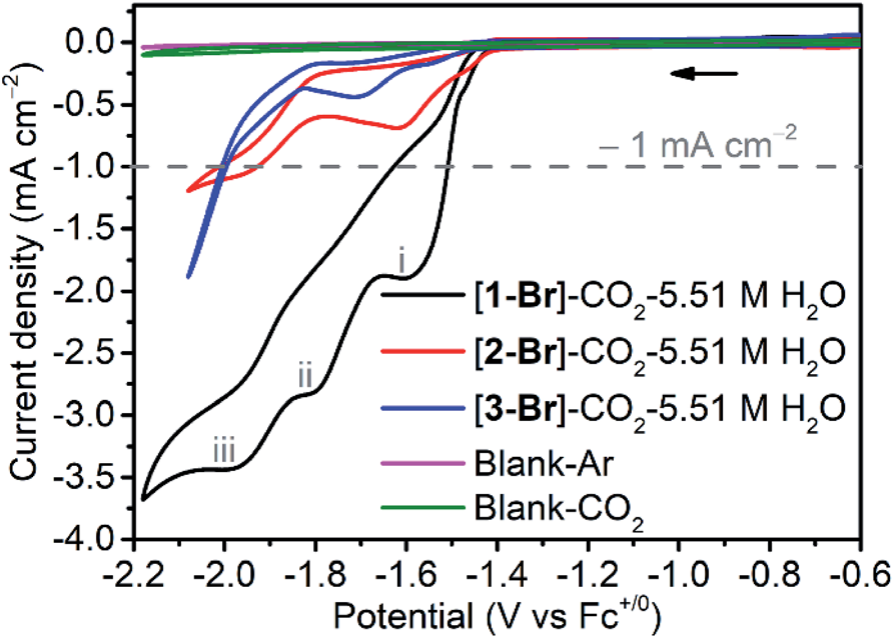
CV measurements of complexes [**1-Br**], [**2-Br**] and [**3-Br**] under CO_2_ with 5.51 M H_2_O added in CH_3_CN solution. The dashed line indicates the current density level as −1.0 mA cm^−2^. The three catalytic waves correspond to (i) the dimer, (ii) the *protonation-first* and (iii) the *reduction-first* pathways.

In CO_2_-saturated dry acetonitrile, a minor change in the reduction peaks (Fig. S20b†) induced by redox-silent solvolysis of Br^−^ to MeCN-bound species is observed, while the addition of H_2_O induced a strong enhancement of the cathodic current (Fig. 2 and S29†). The current increase corresponds to the electrocatalytic reduction of CO_2_ to CO, as verified by controlled potential electrolysis (CPE) experiments (*vide infra*). As shown in Fig. S29,† when the concentration of H_2_O was increased to 5.51 M, the complex [**1-MeCN**]^+^ exhibited the optimal electrocatalytic performance, and three catalytic waves were observed at *ca.* −1.55, −1.85 and −2.05 V. This phenomenon is different from the catalytic properties of published [*fac*-Mn(bpy-R)(CO)_3_Br] complexes, which have one or two catalytic waves. For those that displayed only one catalytic wave, Kubiak, Carter and their co-workers proposed a *reduction-first pathway*,^7,47^ where the metallocarboxylic acid intermediate *fac*-Mn(bpy-R)(CO)_3_(COOH) generated through the reaction between the two-electron reduced anionic species [*fac*-Mn(bpy-R)(CO)_3_]^−^ and CO_2_ was reduced firstly (reduction-first step) and the protonation of the carboxylic acid group in the second step promotes the C–OH bond cleavage. Once the second coordination sphere effects are introduced into the bpy ligand, some of the [*fac*-Mn(bpy-R)(CO)_3_Br] complexes start to exhibit two catalytic waves. The waves at low and high overpotential are assigned to the *protonation-first* and *reduction-first pathways* respectively as theoretically predicted by Carter and experimentally corroborated by Rochford, Grills and Ertem.^41,47^ In the *protonation-first pathway*, the protonation of the metallocarboxylic acid intermediate *fac*-Mn(bpy-R)(CO)_3_(COOH) occurs first followed by H_2_O evolution *via* the second coordination sphere through weak hydrogen bonding; the reduction of the resulting tetracarbonyl intermediate *fac*-Mn(bpy-R)(CO)_4_, as the second step, leads to fast dissociation of CO and the regeneration of the catalyst. Interestingly, the complex [**1-MeCN**]^+^ with a local amide –NHMe group in the secondary coordination sphere displayed three catalytic waves at *ca.* −1.55, −1.85 and −2.05 V (Fig. 2). On the basis of theoretical calculations, the two catalytic waves at −1.85 and −2.05 V are proposed to be the *protonation-first* pathway and the *reduction-first* pathway, respectively (see the Computational section below for more details). The first catalytic wave was proposed to follow the dimer pathway. As shown in Fig. S30,† before the first catalytic wave there is a small reduction wave, which overlapped fully with the first reduction wave of the complex [**1-MeCN**]^+^ under Ar conditions, inducing the production of the [**12**]^0^ dimer. Apparently, the first catalytic wave at −1.55 V is related to the dimer complex [**12**]^0^, which is analyzed in detail in the Computational section. Different from the complex [**1-MeCN**]^+^, complexes [**2-MeCN**]^+^ and [**3-MeCN**]^+^ without any local amide –NHMe group in the secondary coordination sphere show a weak catalytic activity at around −1.6 V (*dimer* pathway) and one moderate catalytic wave at around −2.0 V (*reduction-first* pathway) under CO_2_ with 5.51 M H_2_O added (Fig. 2, S21b and S22b†).

In order to validate the electrochemical CO_2_ reduction ability of [**1-MeCN**]^+^ (*the real form of the pre-catalyst [***1-Br***]* in solution) as observed in the CV measurements, controlled potential electrolysis (CPE) was carried out under different applied potentials. Firstly, a 2 hour CPE experiment (Fig. S31†) was performed at an applied potential of *E*_app_ = −1.55 V in CO_2_ saturated CH_3_CN solution (5.51 M H_2_O). Gas chromatography (GC) analysis shows that CO was the main product during the 2 h electrolysis with a high faradaic efficiency FE_CO_ ≈ 90%, and no hydrogen gas was detected. A charge of 7.24 C passed over 2 h, corresponding to a TON_CO_ value of 7.5. Our results clearly show that [**1-MeCN**]^+^ could catalyze electrochemical CO_2_ reduction at low overpotential. The main catalytic product in the second catalytic wave (*E*_app_ = −1.85 V) is also CO with FE_CO_ ≈ 90% (Fig. S32†). A charge of 19.8 C passed over 2 h and the associated TON_co_ is 19. The CPE experiment at *E*_app_ = −2.05 V also produces CO as the sole gas product with FE_CO_ ≈ 93% (Fig. S33†). A charge of 30.5 C passed over 2 h, corresponding to a TON_CO_ value of 32. Additionally, CPE experiments were also performed with complexes [**2-MeCN**]^+^ and [**3-MeCN**]^+^. As shown in Fig. S34,† the currents of complexes [**2-MeCN**]^+^ and [**3-MeCN**]^+^ under an applied potential of *E*_app_ = −1.85 V in Ar and CO_2_ saturated CH_3_CN solution (5.51 M H_2_O) are almost overlapped with each other and no CO gas was detected by GC analysis. Under an applied potential of *E*_app_ = −2.05 V, GC analysis shows that CO was the main product during the 2 h electrolysis and the faradaic efficiency, FE_CO_, of complexes [**2-MeCN**]^+^ and [**3-MeCN**]^+^ is 87% and 92%, respectively (Fig. S35 and S36†). The consumed charge of complexes [**2-MeCN**]^+^ and [**3-MeCN**]^+^ over 2 h is 5.2 and 14.5 C, corresponding to the TON_CO_ values of 5.4 and 16.1. The consumed charge of the complex [**1-MeCN**]^+^ is respectively 6.0 and 2.2 times larger than that of complexes [**2-MeCN**]^+^ and [**3-MeCN**]^+^ under the same conditions. These results verified that [**1-MeCN**]^+^ with a local amide –NHMe group in the secondary coordination sphere catalyzes electrochemical CO_2_ reduction more effectively than complexes [**2-MeCN**]^+^ and [**3-MeCN**]^+^.

It is also worth noting that the catalytic current of [**1-MeCN**]^+^ starts to increase from −1.5 V (Fig. 2). To the best of our knowledge, [**1-MeCN**]^+^ is the most effective Mn-based CO_2_ reduction pre-catalyst with low over-potential using water as the proton source (Table S8†). Overpotential is defined as the difference between the catalytic potential and the equilibrium potential. Appel and Helm suggested the use of the potential at half of the catalytic current as the catalytic potential, denoted as *E*_cat/2_.^48^ Then the catalytic potentials for the three catalytic waves of [**1-MeCN**]^+^ are −1.51, −1.73 and −1.91 V. Matsubara experimentally estimated the standard electrode potential (*E*^0^) for the reduction of CO_2_ to CO in an acetonitrile–water mixture and showed that the equilibrium potential (*E*_eq_) for the electrochemical reduction of CO_2_ to CO can be formulated as:^49^1
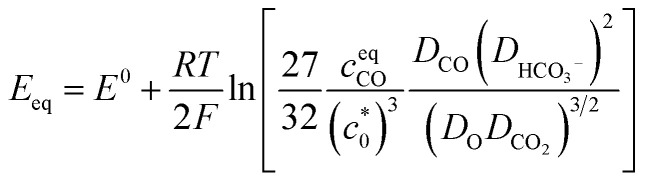
where *F* is the Faraday constant, *R* is the gas constant, *T* is the temperature, *c*^eq^_CO_ is the concentration of CO in the solution with CO at 1 bar in the gas phase, 
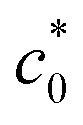
 is the concentration of the catalyst, and *D*_CO_ (2.2 × 10^−5^ cm^2^ s^−1^),^50^
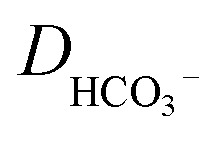
 (1.0 × 10^−5^ cm^2^ s^−1^),^51^*D*_CO2_ (2.0 × 10^−5^ cm^2^ s^−1^)^51^ and *D*_O_ (0.5 × 10^−5^ cm^2^ s^−1^)^52^ are the diffusion coefficients of CO, HCO_3_^−^, CO_2_ and the catalyst, respectively. *E*^0^ under CO_2_-saturated CH_3_CN with 5.51 M H_2_O is −1.37 V *vs.* Fc^+/0^.^49^*E*_eq_ = −1.17 V *vs.* Fc^+/0^ is then obtained for the complex [**1-MeCN**]^+^ in CH_3_CN solution with 5.51 M H_2_O. Accordingly, we obtained the overpotentials associated with these catalytic waves as 0.34, 0.56 and 0.74 V, respectively. By the same methods, the equilibrium potentials of complexes [**2-MeCN**]^+^ and [**3-MeCN**]^+^ and other published Mn-based catalysts were obtained. Because not all catalytic potentials (*E*_cat/2_) of these catalysts could be accurately obtained, we compared the applied potential at a catalytic current density of −1.0 mA cm^−2^. The catalytic data of several Mn-based electrocatalysts together with our complexes have been summarized in Table S8.† The applied potential of the complex [**1-MeCN**]^+^ is −1.51 V, and complexes [**2-MeCN**]^+^ and [**3-MeCN**]^+^ require more negative applied potentials than [**1-MeCN**]^+^ by 0.42 and 0.50 V, respectively. Overall, [**1-MeCN**]^+^ requires the lowest applied potential among these catalysts to reach a current density of −1.0 mA cm^−2^. The local amide –NHMe group as the second coordination sphere effectively promotes the reduction of CO_2_ for the complex [**1-MeCN**]^+^ at low overpotential.

To obtain the kinetics of the three catalytic waves of [**1-MeCN**]^+^, the CVs of [**1-MeCN**]^+^ at various concentrations and scan rates were recorded under catalytic conditions. The catalytic current of all three waves increased linearly with the concentration of [**1-MeCN**]^+^ (Fig. S37†). In addition, the current density of all three catalytic waves showed scan rate dependence in the range of *v* = 0.1 to 1.4 V s^−1^ (Fig. S38†); as the scan rate further increases, the catalytic current plateaus of the three waves are relatively scan rate independent in the range of *v* = 1.4–1.8 V s^−1^. The plot of *i*_cat_/*i*_p_*versus* inverse square root of the scan rate highlights that steady-state conditions are accomplished at high scan rates over 1.4 V s^−1^. These results implied that the pure kinetic regime was approached by increasing the scan rate to 1.4 V s^−1^.^41^ As shown in Fig. S38b–d,† the TOF_max_ of the third (246 ± 1 s^−1^), second (170 ± 4 s^−1^) and first (79 ± 2 s^−1^) catalytic processes is calculated using eqn (2) with the data obtained at 1.4, 1.6 and 1.8 V s^−1^:^53,54^2
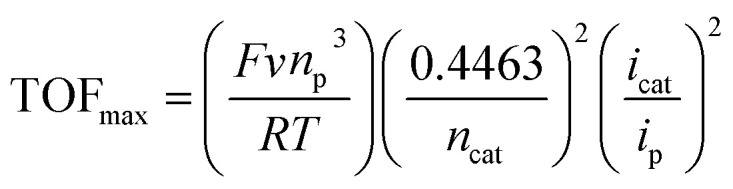
where *F* is the Faraday constant, *R* is the gas constant, *T* is the temperature, *v* is the scan rate, *n*_p_ is the number of electrons involved in the non-catalytic faradaic process (1 electron for [**1-MeCN**]^+^), *n*_cat_ is the number of electrons required for a single catalytic cycle (2 electrons for CO_2_ to CO), and *i*_cat_/*i*_p_ is the ratio of the catalytic current and the non-catalytic faradaic peak current.

### Characterization of reaction intermediates

Fourier-transform infrared spectroelectrochemistry (FTIR-SEC) of [**1-MeCN**]^+^ together with computed *ν*_CO_ bands of several intermediates (Table S9†) were used to identify the reduced species in CH_3_CN solution with 5.51 M H_2_O under electrochemical conditions. FTIR-SEC spectra under an Ar atmosphere at different applied potentials are shown in Fig. S39–S43.† [**1-MeCN**]^+^ shows three *ν*_CO_ bands at 2025, 1932, and 1926 cm^−1^. At an applied potential of −1.55 V, the bands of [**1-MeCN**]^+^ started to fade away and new CO bands at 1978, 1932 and 1865 (br) cm^−1^ (Fig. S40a,† labelled red cycles) were produced and the new species is assigned to the dimeric complex of [**12**]^0^. The reduction of [**3-MeCN**]^+^ is known to produce a dimeric complex [**32**]^0^. [**32**]^0^ was then electrochemically generated (Fig. S41a†) and its FTIR spectrum was plotted together with that of [**12**]^0^ (Fig. S42†). The complex [**32**]^0^ has four *ν*_CO_ stretching bands at 1975, 1932, 1878 and 1856 cm^−1^, in line with the reported values. In comparison with [**32**]^0^, only three bands were observed for [**12**]^0^, while the broad, lower energy band of [**12**]^0^ very likely consists of two vibration modes that are too close to separate. Further decrease of the applied potential leads to the growth of a set of new CO bands at 1917 and 1818 (br) cm^−1^ (Fig. S40b†), assigned to the doubly reduced [**1**]^−^ species. These conclusions are further corroborated by the computed *ν*_CO_ bands (Table S9†). The doubly reduced [**2**]^−^ and previously reported [**3**]^−^ species also have similar CO bands (Fig. S43b and S41b†). The singly and doubly reduced species of [**1-MeCN**]^+^ can also be prepared *via* chemical reduction with KC_8_. As shown in Fig. S44a,† the CO bands of singly and doubly reduced species by KC_8_ are very similar to the CO bands observed in the FTIR-SEC spectra, except that the band at 1817 cm^−1^ of [**1**]^−^ generated in the FTIR-SEC experiment splits into two CO bands at 1826 and 1800 cm^−1^ for the chemically generated [**1**]^−^ in the solution without TBAP. As depicted in Fig. S44b,† a comparison of the FTIR spectra of chemically generated [**1**]^−^ in the THF solutions in the presence and absence of TBAP confirmed that TBAP indeed induced the shift of CO bands.

**Fig. 3:**
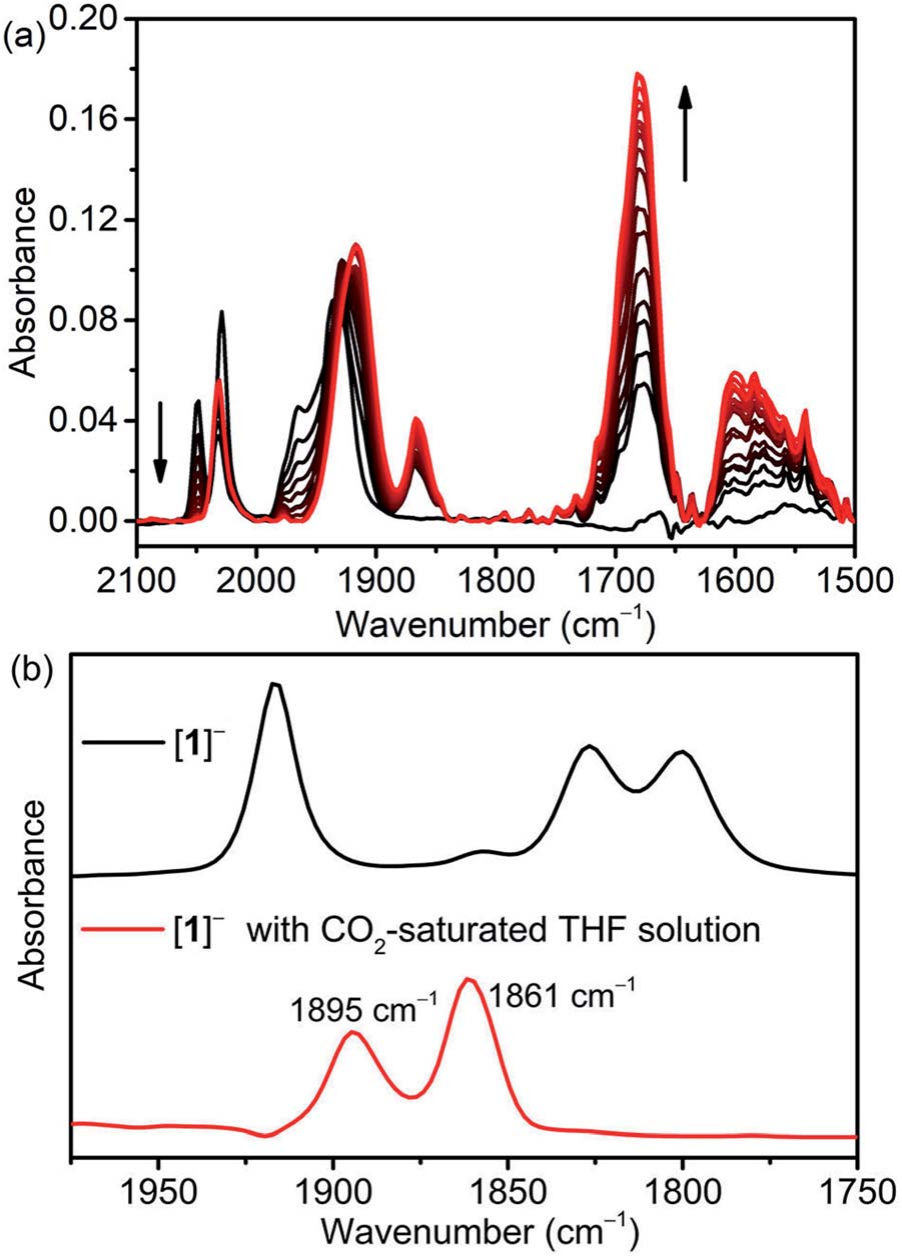
(a) FTIR-SEC changes observed during the reaction (applied potential −1.75 V) of [**1-MeCN**]^+^ (5 mM) in CH_3_CN solution (0.05 M TBAP and 5.51 M H_2_O) under CO_2_. Black and red curves represent the starting and the final spectra, respectively; (b) FTIR spectra of [**1**]^−^ (black) and [**1**]^−^ in CO_2_-saturated THF solution (red).

**Fig. 4:**
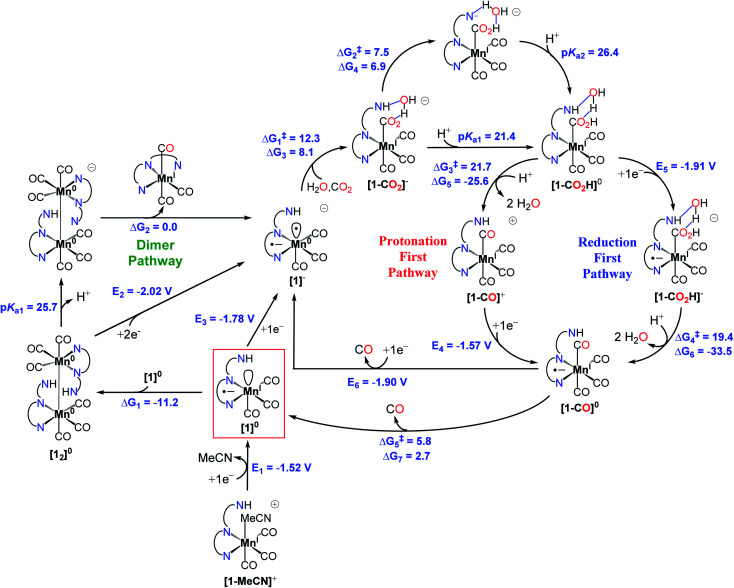
Proposed mechanism for the reduction of CO_2_ to CO by the pre-catalyst [**1-MeCN**]^+^.

In a CO_2_-saturated CH_3_CN solution (5.51 M H_2_O) of [**1-MeCN**]^+^ and at an applied potential of −1.75 V, the CO bands of [**1-MeCN**]^+^ decreased in intensity and a new set of CO bands at 2033, 1916 (br), 1867, 1680 and 1596 cm^−1^ appeared (Fig. 3a), and the corresponding differential IR spectra (Fig. S45†) confirmed the formation of new species. The doubly reduced species [**1**]^−^ is not observed. The strong vibrational band at 1680 cm^−1^ is assigned to free HCO_3_^−^.^10^ The rest of the new bands are tentatively assigned to the metallocarboxylic acid species; specifically, the broad band at 1596 cm^−1^ is due to the vibration of the Mn–COOH group. Kubiak and co-authors have reported the Re–COOH vibration band at 1616 cm^−1^, the value of which is very similar to our observation for the Mn–COOH species.^55^ Gibson and co-authors have also reported the vibration of the Re–COOH group at 1572 cm^−1^.^56^ On the other hand, it's not straightforward to make an assignment based on the computed *ν*_CO_ bands, but among them only [**1-CO2**]^−^ and [*fac*-Mn(bpy-CONMe)(CO)_2_(CO_2_H)]^−^ exhibit low energy bands in the 1640–1650 cm^−1^ region together with overlapping bands with those of bicarbonates/carbonates. To further probe the assignment of this metallocarboxylic acid, [**1**]^−^ was chemically generated by the reaction of [**1-Br**] and KC_8_, and then reacted with CO_2_. The color of the [**1**]^−^ solution changed from blue to brown upon the addition of CO_2_-saturated THF solution, and the corresponding FTIR spectral change is shown in Fig. 3b. The CO bands at 1895 and 1861 cm^−1^ are close to the new set of CO bands at 1915 and 1867 cm^−1^ in the FTIR-SEC experiment (Fig. 3a) and the small difference is caused by the solvation effect (see the FTIR-SEC spectra of the complex [**1-Br**] in THF solution as depicted in Fig. S46†). Finally, the reaction mixture generated by the reaction of [**1**]^−^ and CO_2_ under dry conditions was analyzed by HR-MS, and apparently many peaks were detected (Fig. S47†). The isotope distribution pattern at *m*/*z*^−^ = 396.0029, 397.0042 and 398.0051 fits well with the calculated isotope distribution pattern of the mass fragment [**1 + CO2**]^−^ (Fig. S48†). This mass fragment could be generated from three different species: (i) the metallocarboxylate anion [**1-CO2**]^−^; (ii) the metallocarboxylic acid [**1-COOH**] *via* the deprotonation of carboxylic acid; (iii) the metallocarboxylic acid [**1-COOH**] *via* the deprotonation of the amide group (Fig. 4). Nevertheless, the formation of metallocarboxylic acid or metallocarboxylate species after the reaction of [**1**]^−^ and CO_2_ was presumed to occur as a transient intermediate during catalysis.

### Computational studies

We performed density functional theory (DFT) calculations at the M06 level of theory^57^ in conjunction with the SMD continuum solvation model for acetonitrile^58^ as solvent to investigate the catalytic CO_2_ reduction mechanism of the complex [**1-MeCN**]^+^. On the basis of experimental observations and theoretical calculations coupled with previous mechanistic studies of other Mn-based CO_2_ reduction catalysts,^12,59^ we propose the reaction mechanism depicted in Fig. 4, and further details on Computational methods, activation of the catalyst (Fig. S49†) and computed reaction mechanism (Fig. S50–S56†) are provided in the ESI.† The computed reaction mechanism features three main pathways, namely *dimer*, *protonation-first* and *reduction-first* pathways proposed to occur at the corresponding catalytic waves at −1.55, −1.85 and −2.05 V respectively in the presence of H_2_O as the Brønsted acid.

#### Dimer pathway (*E* = −1.55 V)

The proposed mechanism starts with the one-electron reduction of [**1-MeCN**]^+^ to generate the pentacoordinate [**1**]^0^ species with *E*_1_ = −1.52 V, which is expected to dimerize to generate [**12**]^0^ with Δ*G*_1_ = −11.2 kcal mol^−1^. Based on the observed catalytic wave at −1.55 V, we considered binding of CO_2_ directly to [**1**]^0^ in the presence of H_2_O but the energy requirement for the formation of [**1-CO2H**]^+^, formally a *Mn*^*II*^*–COOH* complex, was found to be prohibitively high (Δ*G* ≈ 50.0 kcal mol^−1^, Fig. S50†). The unfavourable energetics of CO_2_ binding to [**1**]^0^ also diminishes the possibility of formation of CO_2_ sandwiched dimer species [**1-CO2-1**]^0^ (Fig. S50†), as proposed for the Re(bpy)(CO)_3_ class of complexes.^60^ Next, we considered possible CO_2_ activation pathways starting from the [**12**]^0^ dimer and found dissociation of a CO molecule to generate a vacant site on one of the Mn centers to be energetically more accessible (Δ*G* = 22.2 kcal mol^−1^) compared to [**32**]^0^ (Δ*G* = 38.1 kcal mol^−1^, Fig. S52†), in part due to the coordination of the amido group in [**12**]^0^ to the metal center upon the dissociation of CO (the product is denoted as [**12**]^0^-**CO**). However, the optimized TS structures for CO dissociation involve significantly high activation free energies (Δ*G*^‡^ = 38.3 kcal mol^−1^, Fig. S51†), indicating that this pathway is quite unlikely. We still performed an exhaustive search for possible conformers resulting from CO_2_ binding to [**12**]^0^-**CO** species and found the located structures to be quite high in energy (Δ*G* ≈ 40.0 to 60.0 kcal mol^−1^, Fig. S52†), so that the formation of *mer*-[**1-CO2H**]^+^ from such intermediates is not plausible. On the other hand, the deprotonation of one of the amide groups in [**12**]^0^ is possible (p*K*_a_ = 27.8) and becomes more favorable in the presence of H_2_O (p*K*_a1_ = 25.7) (Fig. 4 and S53†). Thus, we propose that deprotonation leads to stronger coordination of the amide N to the Mn center leading to the destabilization of the dimer and a consequent disproportionation to generate [Mn(bpy-CONMe)(CO)_3_] and [**1**]^−^ (Δ*G*_2_ = 0.0 kcal mol^−1^), the latter of which can follow the *protonation first* pathway (described below) at −1.55 V for the catalytic reduction of CO_2_ to CO. Although other alternative pathways could not be excluded, the proposed reaction mechanism seems to be the most plausible among the alternatives considered.

#### Protonation-first pathway (*E* = −1.85 V)

Further reduction of [**1**]^0^ (*E*_3_ = −1.78 V) (or the disproportionation reaction mentioned above in the *dimer* pathway) generates the active catalytic species [**1**]^−^. In the presence of H_2_O as a weak Brønsted acid, the –NHMe group acts as a hydrogen bond/proton donor to assist the next step of CO_2_ binding to the doubly reduced [**1**]^−^ intermediate to form [**1-CO2**]^−^ (Δ*G*^‡^_1_ = 12.3 and Δ*G*_3_ = 8.1 kcal mol^−1^) and the following proton transfer to the newly formed carboxylate to generate the [Mn(bpy-CONMe)(CO)_3_(CO_2_H)]^−^ intermediate with a deprotonated amide group (Δ*G*^‡^_2_ = 7.5 and Δ*G*_4_ = 6.9 kcal mol^−1^) (Fig. S54†). Further protonation of this intermediate (p*K*_a2_ = 26.4) generates [**1-CO2H**]^0^. The formation of CO and H_2_O from the [**1-CO2H**]^0^ intermediate could proceed *via* two possible routes labeled *protonation-first* and *reduction-first* pathways (Fig. 4). The *protonation-first pathway* starts with heterolytic C–OH bond cleavage, leading to the formation of H_2_O and [**1-CO2**]^+^ (Δ*G*^‡^_3_ = 21.7 and Δ*G*_5_ = −25.6 kcal mol^−1^), which is further reduced to generate [**1-CO**]^0^ (*E*_4_ = −1.57 V).

#### Reduction-first pathway (*E* = −2.05 V)

In contrast, the *reduction-first pathway* starts with the reduction of [**1-CO2H**]^0^ (*E*_5_ = −1.91 V) followed by C–OH bond cleavage (Δ*G*^‡^_4_ = 19.4 and Δ*G*_6_ = −33.5 kcal mol^−1^) (Fig. S55†), yielding H_2_O and the common [**1-CO**]^0^ intermediate. Next, either (i) CO could evolve from [**1-CO**]^0^ (Δ*G*^‡^_5_ = 5.8 and Δ*G*_7_ = 2.7 kcal mol^−1^) to generate [**1**]^0^ or further reduction of [**1-CO**]^0^ results in the spontaneous dissociation of CO to generate the active catalytic intermediate [**1**]^−^ (*E*_6_ = −1.90 V).

**Fig. 5:**
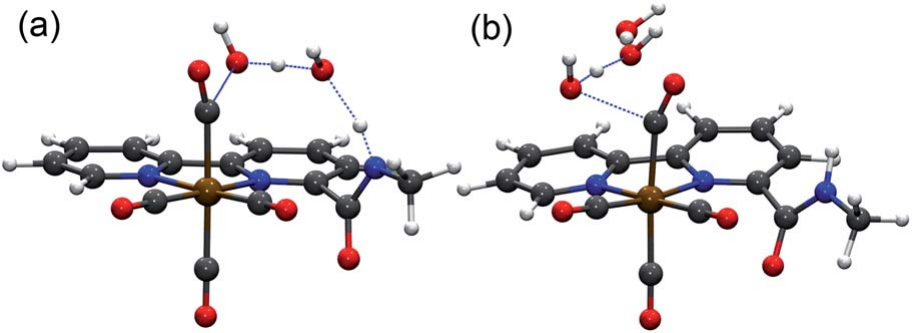
Optimized transition state structures for C–OH bond cleavage in [**1-CO2H**]^0^ with (a) and without (b) the assistance of the amide group in the second coordination sphere and H_2_O as the acid.

The theoretical calculations demonstrate the critical role of the –NHMe group in the second coordination sphere for the destabilization of the dimer [**12**]^0^ species upon deprotonation, leading to the disproportionation reaction as well as the stabilization of the transition state of the rate-limiting chemical step of heterolytic C–OH bond cleavage. In the presence of H_2_O as a weak Brønsted acid, the –NHMe group acts as a hydrogen bond and proton donor in the second coordination sphere leading to a Δ*G*^‡^_3_ of 21.7 kcal mol^−1^ for the C–OH bond cleavage step *via* the *protonation-first* pathway (Fig. 5a), whereas the absence of hydrogen bonding stabilization from the –NHMe group results in an increase of approximately 7 kcal mol^−1^ in activation free energy, Δ*G*^‡^ = 28.5 kcal mol^−1^ (Fig. 5b), a value quite similar to that computed for the optimized TS structure of complex **3** (Fig. S56,† Δ*G*^‡^ = 26.3 kcal mol^−1^, see the ESI† for further details). This indicates the essential role of the –NHMe group in effectively reducing the energy requirement to perform C–OH bond cleavage and promoting the *dimer* and *protonation-first* pathways and thereby providing access to a low overpotential route for the catalytic reduction of CO_2_ to CO with H_2_O as a weak Brønsted acid.

## Conclusions

We have reported a Mn-based CO_2_ reduction pre-catalyst [**1-MeCN**]^+^ that bears an amide –NHMe group in the second coordination sphere. The presence of this amide –NHMe group leads to a drastic enhancement in its catalytic activity, especially at mild potentials, using H_2_O as the proton source such that it surpasses the reference pre-catalyst [**2-MeCN**]^+^. Theoretical calculations demonstrate the critical role of the amide proton of the –NHMe group in accelerating C–OH bond cleavage and enabling a low over-potential route for the catalytic reduction of CO_2_ to CO with H_2_O as a weak Brønsted acid *via dimer* and *protonation-first* pathways. The straightforward synthesis of amide functionalized ligands and the good stability of the amide group allow the fine tuning of the catalyst properties. We believe that this work will inspire the scientific community to develop more efficient Mn catalysts and functional materials using amide groups as the second coordination sphere.

## Experimental section

### General considerations

To ensure rigorous air- and water-free conditions, anhydrous acetonitrile (CH_3_CN), tetrahydrofuran (THF) and *n*-hexane were distilled and stored in a glovebox. Distilled THF and *n*-hexane were stored over the Na–K alloy. Tetrabutylammonium hexafluorophosphate (TBAP, Sigma-Aldrich, 98%) and 18-crown-6 (Aladdin, 98%) were dried under vacuum at 100 °C for 12 h. Potassium graphite^61^ (KC_8_) and ligands bpy-CONHMe and bpy-CONMe_2_ (Fig. S1†) were synthesized according to literature methods.^62^ Other reagents were used as received: (2,2′-bipyridine)-6-carboxylic acid (Zhengzhou Alfachem, 98%), NH_2_CH_3_ (Energy Chemical, 2 M in THF), ClCOOC_2_H_5_ (Energy Chemical, 98%), [Mn(CO)_5_Br] (Aldrich, 98%) and Ag(OTf) (Aladdin, 97%).

The NMR spectra were recorded on a Bruker 400 MHz spectrometer. Mass spectrometry was performed on a Q-Exactive. Fourier-transform infrared (FTIR) spectra were collected on a Bruker Alfa. Fourier-transform infrared spectroelectrochemistry (FTIR-SEC) spectra were collected on a Bruker V80 with a home-made IR spectroelectrochemical cell. Elemental analysis was performed by using a Thermoquest-Flash EA 1112 elemental analyzer for C, H, and N. The studies of single-crystal X-ray diffraction were performed on a Bruker D8 VENTURE with Mo Kα radiation. The crystals were fixed on a Cryoloop to collect data under a N_2_ stream at a temperature of 100 K. Bruker SAINT software was used to integrate the collected data. The structures of the complexes were produced by using the Olex program. The CCDC numbers for complexes [**1-Br**], [**2-Br**] and [K(18-crown-6)]^+^[**1**]^−^ are 1957948, 1970506 and 1957947, respectively.†

### Synthesis of [*fac*-Mn(bpy-CONHMe)(CO)_3_Br] ([**1-Br**])

The synthetic route of the complex [**1-Br**] is depicted in Fig. S2.† Mn(CO)_5_Br (0.165 g, 1.2 mmol) and bpy-CONHMe (0.107 g, 0.5 mmol) were added into 25 mL of Et_2_O under an Ar atmosphere. The solution was refluxed under dark. After 1 h, the product precipitated from the solution. The mixture was cooled to room temperature, and the orange precipitate was filtered off and washed with 10 mL Et_2_O three times. The solid was dried under vacuum to get [**1-Br**] with a 70% yield. Single crystals of [**1-Br**] were prepared by vapor diffusion of pentane into the THF solution of the complex at −20 °C. ^1^H NMR (400 MHz, DMSO-*d*) *δ* 9.2–9.27 (1H), 9.05–9.18 (1H), 8.6–8.72 (2H), 8.2–8.33 (2H), 7.65–7.75 (2H), 2.8–2.89 (3H); IR (THF) *ν*_CO_ 2025, 1937, 1922, 1682 cm^−1^; elemental analysis calcd (%) for C_15_H_11_N_3_O_3_BrMn: C 43.30, H 2.66, N 10.10; found: 43.34, 2.68, 10.07; ESI-MS (positive mode): [**1**]^+^ 352.01.

### Synthesis of [*fac*-Mn(bpy-CONHMe)(CO)_3_(CH_3_CN)](OTf) ([**1-MeCN**](OTf))

[Mn(CO)_5_Br] (0.275 g, 1.0 mmol) and Ag(OTf) (0.270 g, 1.05 mmol) were added to CH_2_Cl_2_ (25 mL). The mixture was stirred in the dark until all the starting material was converted to [*fac*-Mn(CO)_5_(OTf)] as confirmed by FTIR spectroscopy. The reaction mixture was filtered and the solution was removed by rotary evaporation, yielding a yellow solid. The solid was re-dissolved in diethyl ether (25 mL) and bpy-CONHMe (0.192 g, 0.9 mmol) was then added. The solution was refluxed in the dark overnight, and the precipitate was filtered, washed with 10 mL Et_2_O three times and dried under vacuum, yielding the target complex as a bright yellow solid (0.289 g, 60%). ^1^H NMR (400 MHz, CD_3_CN-*d*) *δ* 9.28–9.35 (1H), 9.20–9.26 (1H), 8.79–8.71 (2H), 8.33–8.48 (2H), 7.80–7.93 (2H), 2.90–2.93 (3H); IR (THF) *ν*_CO_ 2055, 1956, 1940 cm^−1^.

### Chemical reduction of [**1-Br**]

The manganese complex [**1-Br**] (10 mg, 0.023 mmol) was dissolved in THF in an argon-filled glovebox. In order to get singly reduced species ([**12**]^0^), KC_8_ (4 mg, 0.03 mmol) was slowly added into the THF solution and stirred until the IR spectrum of [**1-Br**] completely disappeared. The solution was centrifuged to remove the black solid and a dark red solution of the neutral species was obtained. Due to its instability, an attempt to get the single crystals of this neutral species failed. To prepare the two-electron reduced species [K(18-crown-6)]^+^[**1**]^−^, 18-crown-6 (15 mg, 0.058 mmol) was added into a THF solution of [**1-Br**], and then KC_8_ (7 mg, 0.053 mmol) was gradually added until the CO bands of the complex [**1-Br**] and [**12**]^0^ completely disappeared, resulting in a blue solution of the doubly reduced [**1**]^−^ complex. The single crystals of [K(18-crown-6)]^+^[**1**]^−^ were successfully grown through diffusing pentane into the THF solution of the doubly reduced complex at −20 °C.

### *In situ* reaction of [**1**]^−^ with CO_2_

The CO_2_-saturated THF solution (0.5 mL) was mixed with the THF solution of the doubly reduced complex [**1**]^−^ (1 mL, 0.023 mM under air-free conditions). Then the resulting solution was injected immediately into a Q-Exactive using CH_3_OH/H_2_O (v/v, 1 : 1; not air-free solvents) as an eluent at a capillary temperature of 160 °C.

### Electrochemistry

All electrochemical experiments were carried out with a CHI 760 electrochemical workstation. CV measurements were performed in anhydrous CH_3_CN (5 mL) with 0.1 M TBAP as the supporting electrolyte. Glassy carbon (3 mm) was used as the working electrode, Pt wire as the counter electrode and Ag/AgNO_3_ was used as a reference electrode. Ferrocene is used as an internal standard and all potentials reported herein were converted to the Fc^+/0^ reference scale using *E*(Fc^+/0^) = *E*(Ag/AgNO_3_) − 0.08 V.

Controlled potential electrolysis (CPE) experiments were carried out in a home-made H-type electrochemical cell in which the cathodic and anodic compartments were separated by glass frit. Typical working conditions are as follows: a glass carbon plate as the working electrode, a Pt plate as the counter electrode, Ag/AgNO_3_ as the reference electrode, and 0.1 M TBAP/CH_3_CN with 5.51 M (v/v) H_2_O as the electrolyte. Solutions were saturated with carbon dioxide before electrolysis. Gas analysis for CPE experiments was carried out using an on-line FuLi Instruments GC9790 Plus gas chromatograph with a flame ionization detector under a constant gas flow (20 mL min^−1^; carry gas = Ar).

### Fourier-transform infrared spectroelectrochemistry (FTIR-SEC)

A home-made IR spectroelectrochemical cell was used for this study. The cell consists of a glassy carbon working electrode, a Pt counter electrode, and an Ag pseudo-reference electrode. Ferrocene was used as an internal standard to calibrate the Ag pseudo-reference electrode.

### Computational methods

#### Density functional theory

All geometries were fully optimized at the M06 level of density functional theory^57^ with the SMD continuum solvation model^58^ for acetonitrile as the solvent using the Stuttgart [8s7p6d2f|6s5p3d1f] ECP10MDF contracted pseudopotential basis set^63^ for Mn and the 6-31G(d) basis set^64^ for all other atoms. Non-analytical integrals were evaluated using the integral=grid=ultrafine option as implemented in the Gaussian 16 software package.^65^ The nature of all stationary points was verified by analytic computation of vibrational frequencies, which were also used for the computation of zero-point vibrational energies and molecular partition functions, and for determining the reactants and products associated with each transition-state structure (by following the normal modes associated with imaginary frequencies). Partition functions were used in the computation of 298 K thermal contributions to the free energy employing the usual ideal-gas, rigid-rotator, harmonic oscillator approximation. Free-energy contributions were added to single-point, SMD-solvated M06 electronic energies computed with the optimized geometries obtained with the initial basis with the SDD basis set for Mn and the 6-311+G(2df,p) basis set for all other atoms to arrive at final, composite free energies.

#### Solvation and standard reduction potentials

As mentioned above, solvation effects for acetonitrile were accounted for by using the SMD continuum solvation model. A 1 M standard state was used for all species in solution (except for acetonitrile as solvent for which the standard state was assigned as 19.14 M). Thus, the free energy in solution is computed as the 1 atm gas-phase free energy, plus an adjustment for the 1 atm to the 1 M standard-state concentration change of *RT* ln(24.5), or 1.9 kcal mol^−1^, plus the 1 M to 1 M transfer (solvation) free energy computed from the SMD model. The free energy of solvation of protons in acetonitrile is taken as −260.2 kcal mol^−1^.^66^

Standard reduction potentials were calculated for various possible redox couples to assess the energetic accessibility of different intermediates at various oxidation states. For a redox reaction of the form

3 O_(soln)_ + *n*e_(g)_^−^ → R_(soln)_

where O and R denote the oxidized and reduced states of the redox couple, respectively, and *n* is the number of electrons involved in the redox reaction, the reduction potential *E*^0^_O|R_ relative to the SCE was computed as4
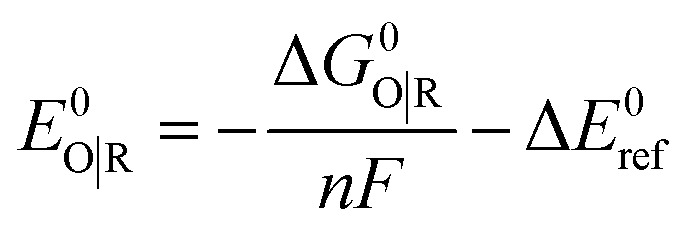
where Δ*G*^0^_O|R_ is the free energy change associated with eqn (3) (using Boltzmann statistics for the electron) and Δ*E*^0^_ref_ is taken as 0.141 V,^67^ which is required for the conversion of calculated *E*^0^_O|R_*versus* the normal hydrogen electrode (NHE) in aqueous solution (*E*_NHE_ = −4.281 V)^68^ to *E*^0^_O|R_*versus* the saturated calomel electrode (SCE) in acetonitrile (*E*_SCE_ = −4.422 V).^69^ We obtained reduction potentials referenced to the ferricenium/ferrocene couple by using a shift of −0.384 V from *E*^0^_O|R_*vs.* SCE.

## Conflicts of interest

There are no conflicts to declare.

## Supplementary Material

SC-012-D0SC05679K-s001

SC-012-D0SC05679K-s002

SC-012-D0SC05679K-s003
