# Supplementary material for: An amide-based second coordination sphere promotes the dimer pathway of Mn-catalyzed CO2-to-CO reduction at low overpotential
Source: Chem Sci. 2021 Feb 11;12(13):4779–88. doi: 10.1039/d0sc05679k (PMC8179605; doi:10.1039/d0sc05679k)
Supplement: SC-012-D0SC05679K-s002 [file SC-012-D0SC05679K-s002.pdf]

Electronic Supplementary Material (ESI) for Chemical Science.
This journal is © The Royal Society of Chemistry 2021

Amide-based second coordination sphere promotes the dimer pathway of Mn-catalyzed CO_2-to-CO reduction at low overpotential

Yong Yang,[a] Mehmed Z. Ertem *[b] and Lele Duan *[a]

[a]Department of Chemistry, Shenzhen Grubbs Institute and Guangdong Provincial Key Laboratory of Energy Materials for Electric Power, Southern University of Science and Technology, Shenzhen, 518055, China

[b]Chemistry Division, Energy & Photon Sciences, Brookhaven National Laboratory, Upton, NY 11973-5000

Ligand exchange study.

It is well known that the axial bromo ligand of [*fac*-Mn(N^N)$(CO)_3$Br] can be partially replaced by CH_3CN in the acetonitrile solution. CVs of [**1**–**Br**] in Ar-saturated dry CH_3CN containing 0.10 M of tetrabutylammonium hexafluorophosphate (TBAP) showed three irreversible reduction waves at −1.51, −1.59 and −1.76 V (Figure S20). The ligand exchange reaction was studied using FTIR spectroscopy in the dry CH_3CN (Figure S26b) and the mixed CH_3CN/H_2O (catalytic conditions; Figure S26a), respectively. Apparently, the solvolysis of [**1**–**Br**] to [**1**–**MeCN**]Br occurs much faster in the mixed acetonitrile/water than in the dry CH_3CN. Nevertheless, a mixture of [**1**–**Br**] and [**1**–**MeCN**]Br was obtained during the time scale of preparing the CV experiments. Additionally, the CV of complex ([**1**–**MeCN**](OTf)) was measured under the same conditions (Figure S24), which displayed only two reduction waves. Accordingly, the first and second reduction waves of [**1**–**Br**] are actually corresponding to the one-electron reduction process of $[\mathbf{1}–\mathbf{MeCN}]^+$ and [**1**–**Br**], respectively. These two peaks could not be separated clearly by varying the scan rate (Figure S25). After one-electron reduction, the resulting Mn^0 species undergoes fast bromo dissociation (EC mechanism), forming 5-coordinate $[Mn^I(bpy\text{-}CONHMe)^{\bullet-}(CO)_3]$ ($[\mathbf{1}]^0$). This $[\mathbf{1}]^0$ monomer is prone to dimerization to yield $[\mathbf{1}_2]^0$ which could be further reduced at E = −1.76 V, leading to the formation of $[Mn^0(bpy\text{-}CONHMe)^{\bullet-}(CO)_3]$ ($[\mathbf{1}]^-$) (CEC mechanism; Figure S20).[1-4] At the reverse scan, the oxidation of $[\mathbf{1}]^0$ to $[\mathbf{1}]^+$ was observed at −1.46 V while the oxidation wave at −0.76 V assigned as the oxidation of $[\mathbf{1}_2]^0$ dimer.[5,6] The scan rate dependence measurements (Figure S25) disclose that although the oxidation waves at −1.46 V (ox1) and −0.76 V (ox2) are both growing upon increasing the scan rate, the ratio of i_{ox1}/i_{ox2} also increased (Table S7) indicating that the dimer formation is inhibited at higher scanning rates. After addition of water to the electrolyte (5.51 M H_2O) under Ar conditions, the reduction wave of [**1**–**Br**] to $[\mathbf{1}–\mathbf{Br}]^0$ at −1.59 V disappeared and only the reduction wave of $[\mathbf{1}–\mathbf{MeCN}]^+$ to $[\mathbf{1}–\mathbf{MeCN}]^0$ remained (Figures S20b), due to the fast ligand exchange in the presence of water.

References

(1) M. D. Sampson, A. D. Nguyen, K. A. Grice, C. E. Moore, A. L. Rheingold and C. P. Kubiak, *J. Am. Chem. Soc.*, 2014, **136**, 5460-5471.

(2) F. Franco, M. F. Pinto, B. Royo and J. Lloret-Fillol, *Angew. Chem. Int. Ed.*, 2018, **57**, 4603-4606.

(3) D. C. Grills, M. Z. Ertem, M. McKinnon, K. T. Ngo and J. Rochford, *Coord. Chem. Rev.*, 2018, **374**, 173-217.

(4) K. T. Ngo, M. McKinnon, B. Mahanti, R. Narayanan, D. C. Grills, M. Z. Ertem and J. Rochford, *J. Am. Chem. Soc.*, 2017, **139**, 2604-2618.

(5) M. Bourrez, F. Molton, S. Chardon-Noblat and A. Deronzier, *Angew. Chem. Int. Ed.*, 2011, **50**, 9903-9906.

(6) J. M. Smieja, M. D. Sampson, K. A. Grice, E. E. Benson, J. D. Froehlich and C. P. Kubiak, *Inorg. Chem.*, 2013, **52**, 2484-2491.

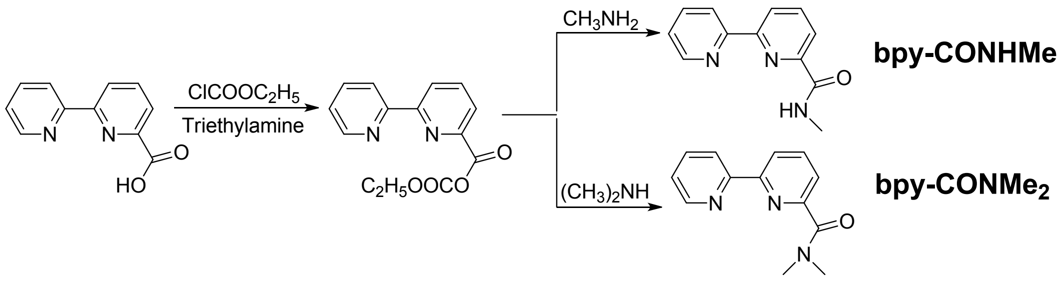

Figure S1. Synthesis of ligands of **bpy-CONHMe** and **bpy-CONMe$_2$**.

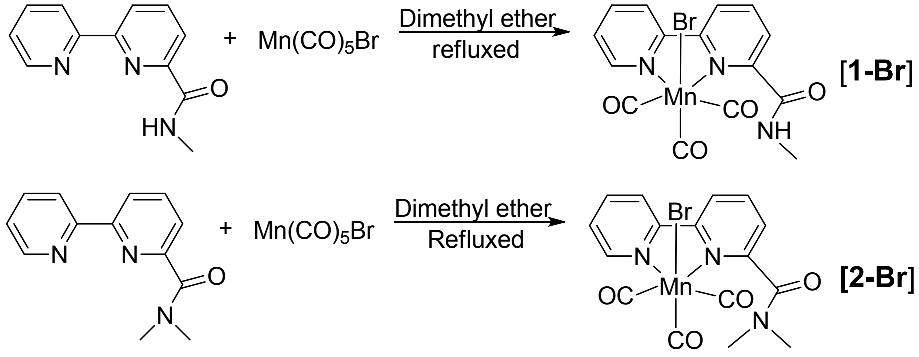

Figure S2. Synthesis of manganese complexes [**1**–**Br**] and [**2**–**Br**].

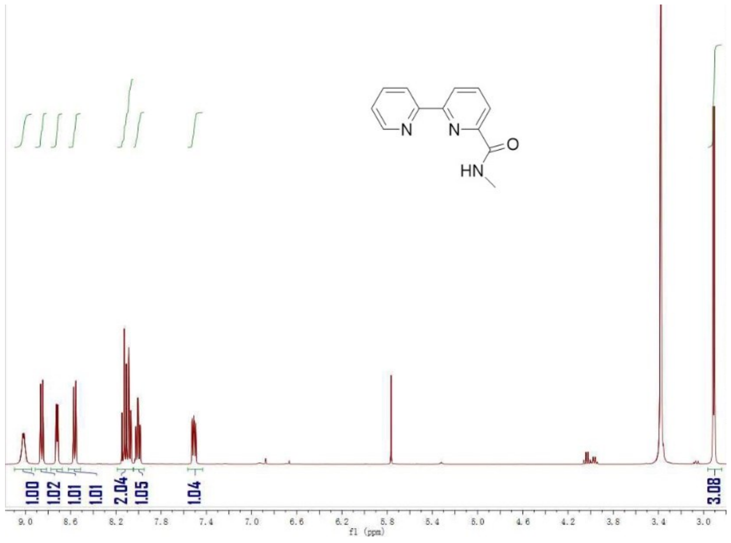

Figure S3. ^{1}H NMR spectrum in DMSO-d$_6$ of ligand **bpy-CONHMe**.

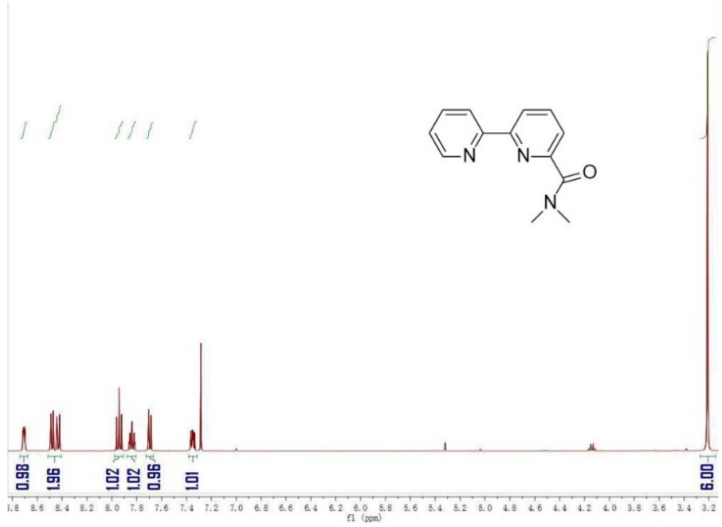

Figure S4. ^{1}H NMR spectrum in $CDCl_3$ of ligand **bpy-CONMe$_2$**.

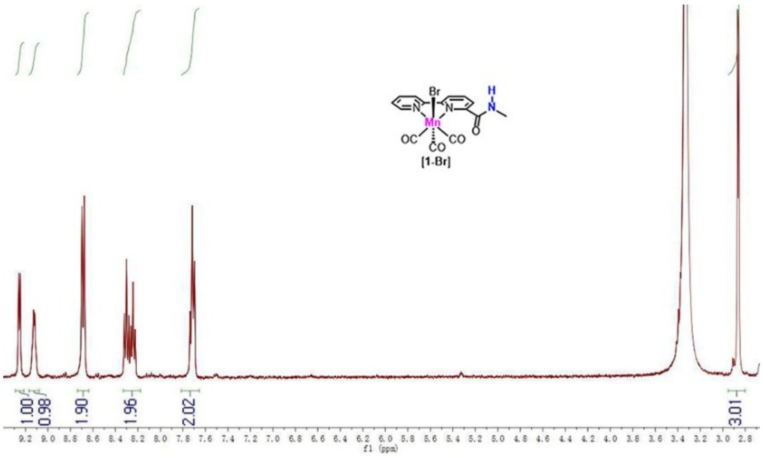

Figure S5. ^{1}H NMR spectrum in DMSO-d$_6$ of complex [**1**–**Br**].

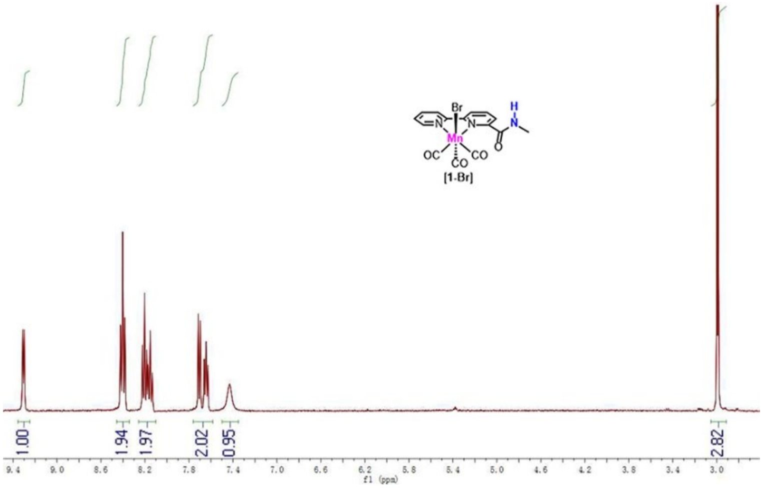

Figure S6. ^{1}H NMR spectrum in CD_3CN of complex [**1**–**Br**].

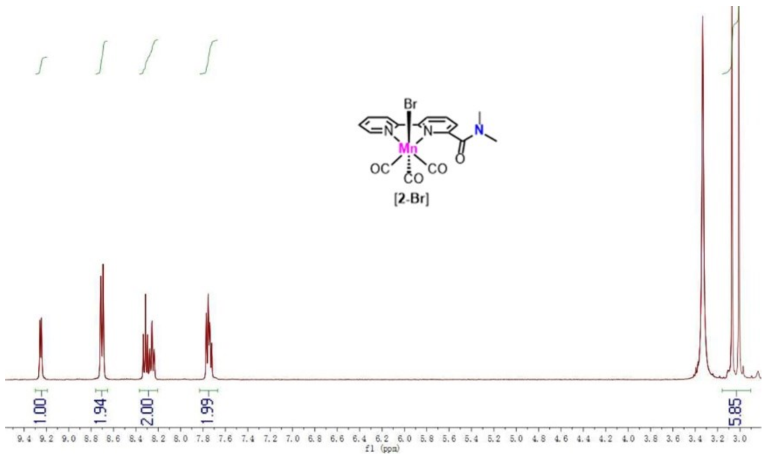

Figure S7. ^{1}H NMR spectrum in DMSO-d_6 of complex [**2**–**Br**].

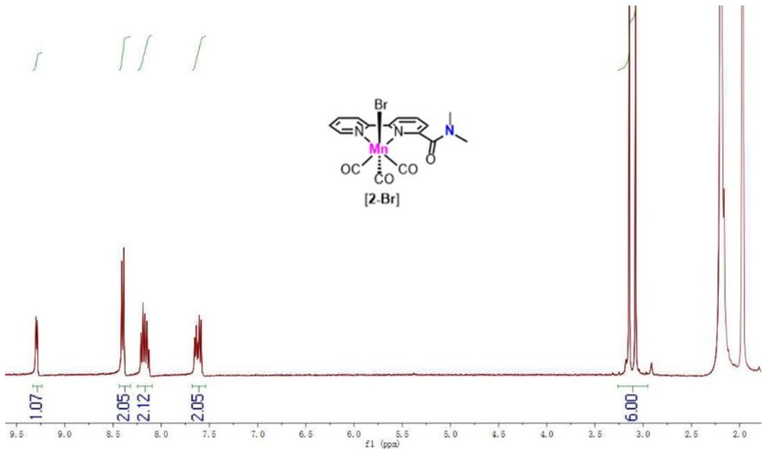

Figure S8. ^{1}H NMR spectrum in CD_3CN of complex [**2**–**Br**].

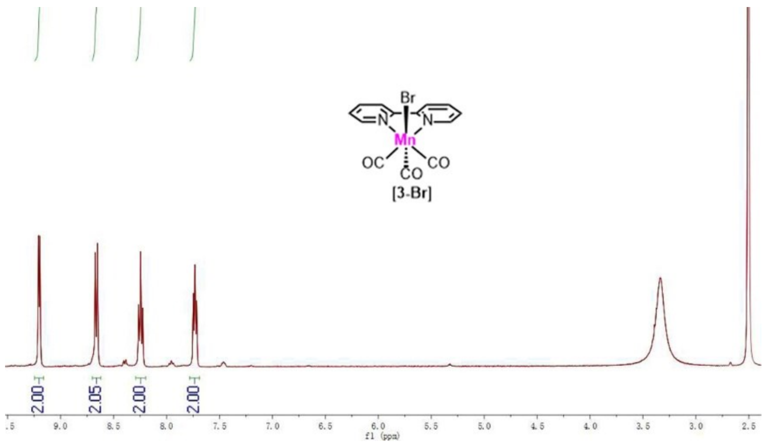

Figure S9. ^{1}H NMR spectrum in DMSO-d_6 of complex [**3**–**Br**].

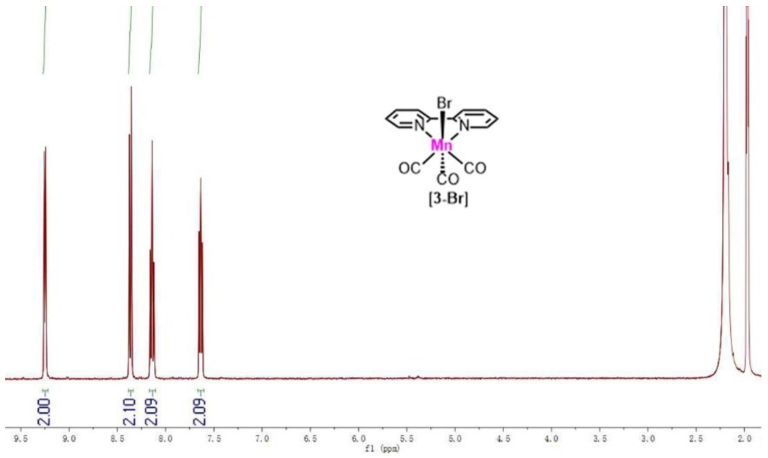

Figure S10. ^1H NMR spectrum in CD_3CN of complex [**3**–**Br**].

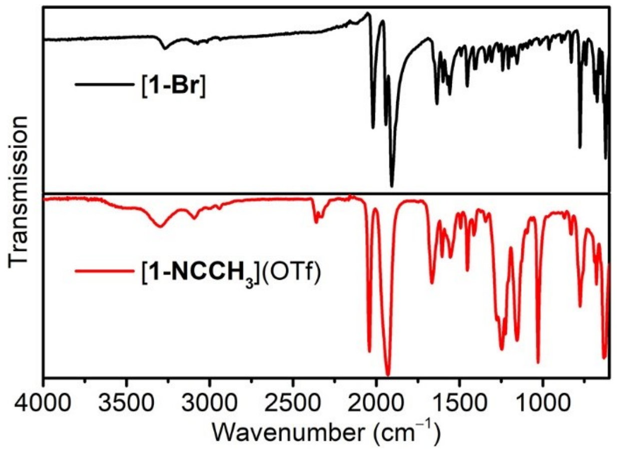

Figure S11. ATR-IR spectra of complexes [**1**–**Br**] and [**1**–**MeCN**](OTf).

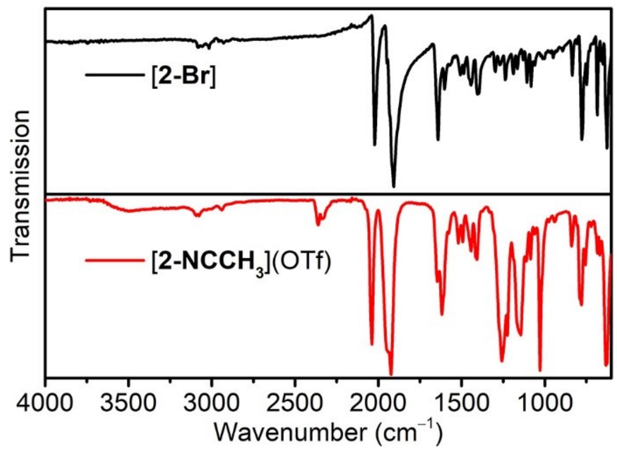

Figure S12. ATR-IR spectra of complexes [**2**–**Br**] and [**2**–**MeCN**](OTf).

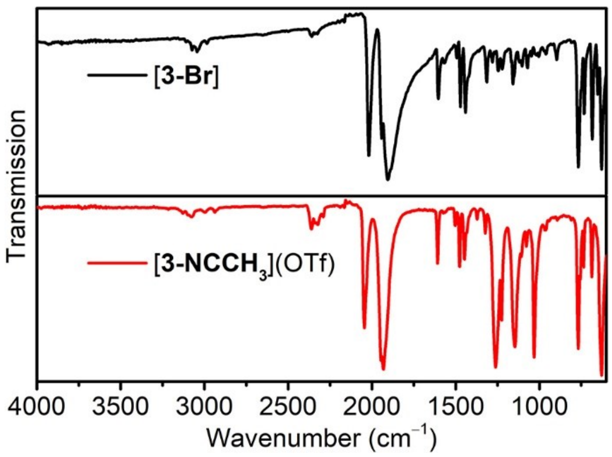

Figure S13. ATR-IR spectra of complexes [**3**–**Br**] and [**3**–**MeCN**](OTf).

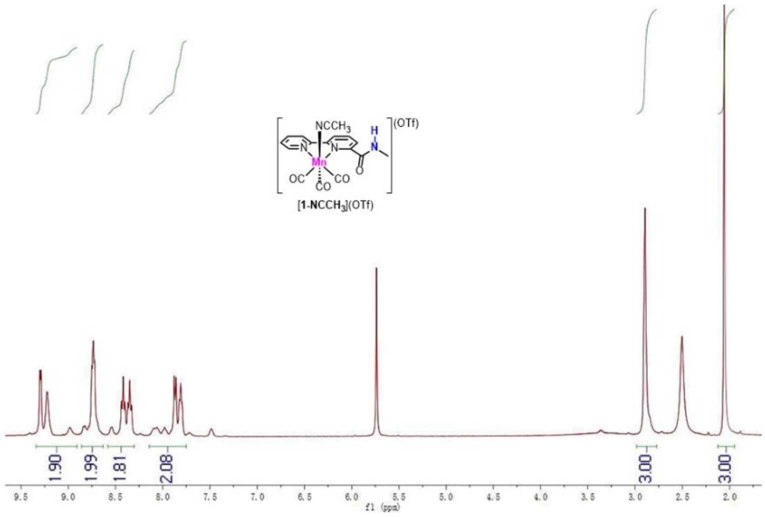

Figure S14. ^{1}H NMR spectrum in DMSO-d$_6$ of complex [**1**–**MeCN**](OTf).

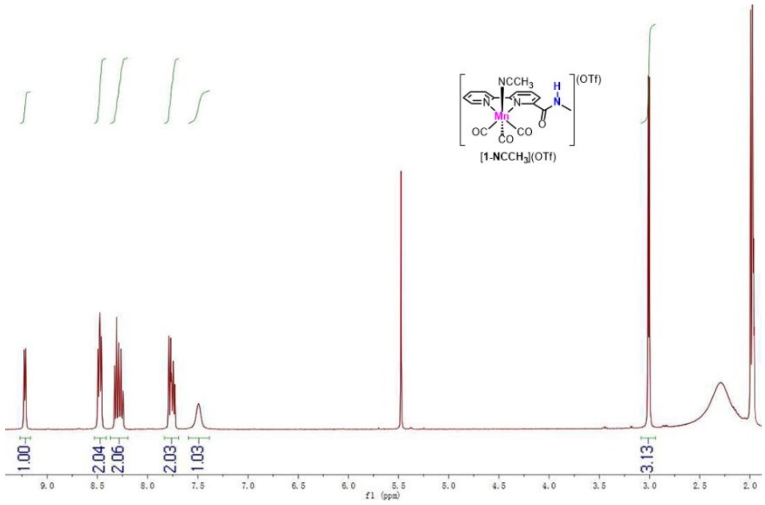

Figure S15. ^{1}H NMR spectrum in CD_3CN of complex [**1**–**MeCN**](OTf).

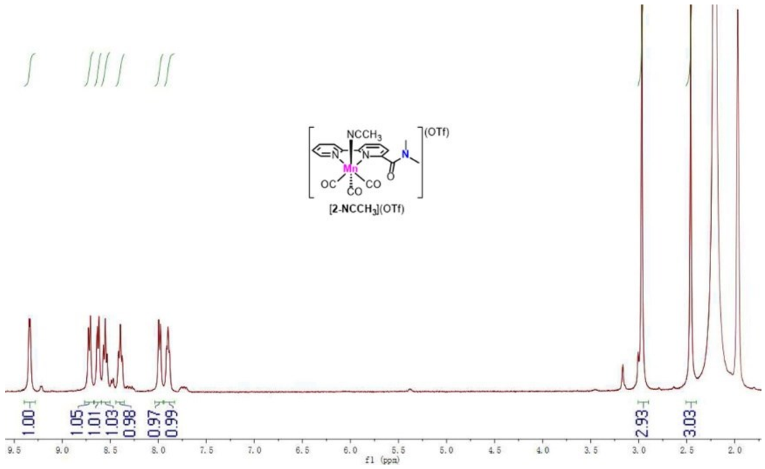

Figure S16. ^{1}H NMR spectrum in CD_3CN of complex [**2**–**MeCN**](OTf).

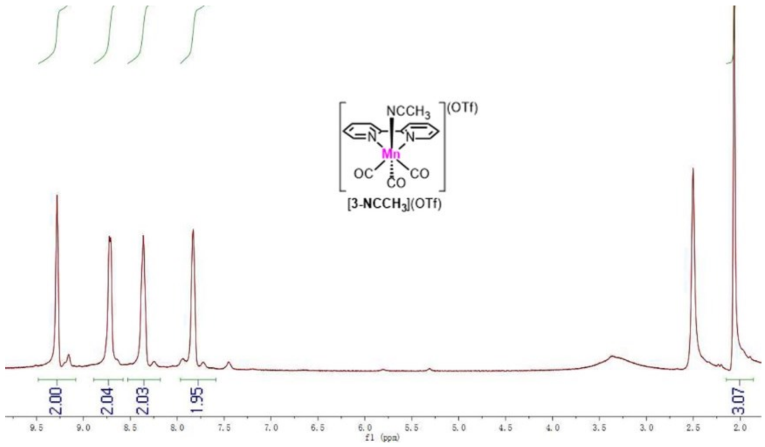

Figure S17. ^{1}H NMR spectrum in DMSO-d_6 of complex [**3**–**MeCN**](OTf).

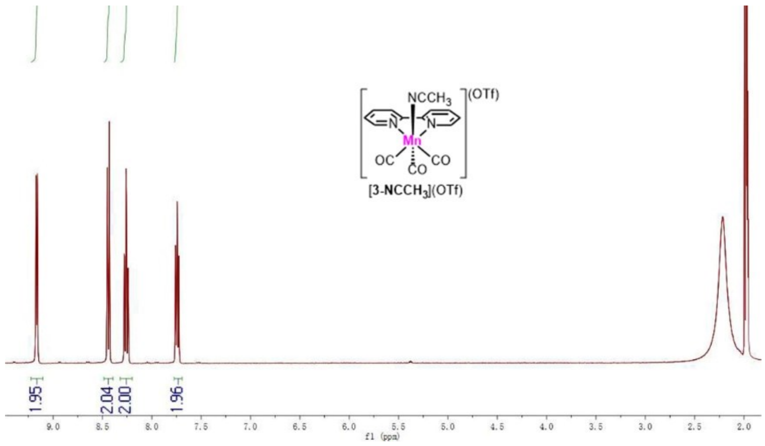

Figure S18. ^{1}H NMR spectrum in CD_3CN of complex [**3**–**MeCN**](OTf).

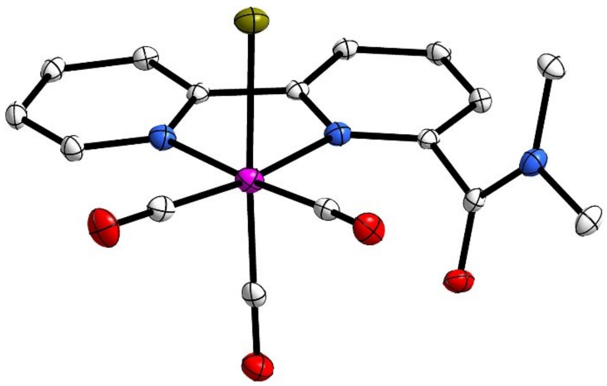

Figure S19. X-ray crystal structure of [**2**–**Br**] with ellipsoids at the 50% probability level.

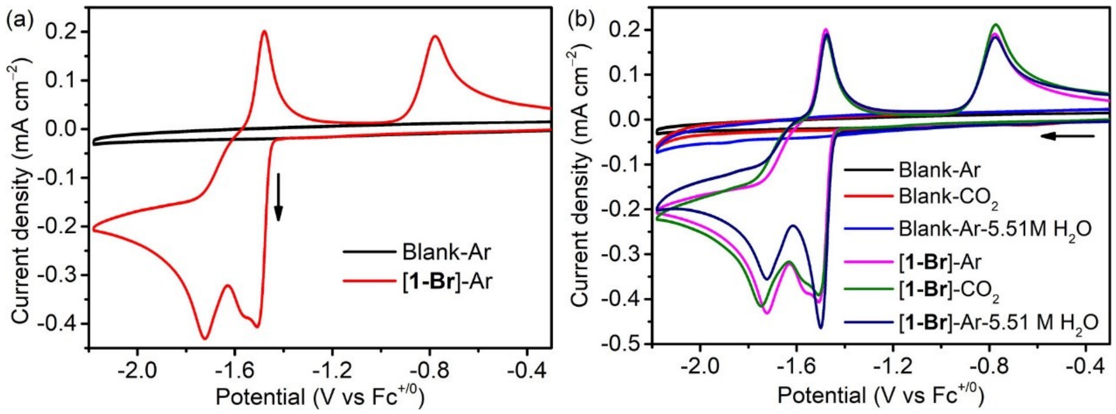

Figure S20. CVs of [**1**–**Br**] (1 mM), under Ar (black), CO_2 (red), Ar with 5.51 M H_2O added (blue) and CO_2 with 5.51 M H_2O added in anhydrous CH_3CN with TBAP (0.1 M) as electrolyte. *Note: It took longer time to prepare the CO_2 saturated solution than the Ar saturated solution, so the concentration of the MeCN-bound species would be higher in the CO_2 saturated solution than the Ar saturated solution, leading to the difference in their CVs.*

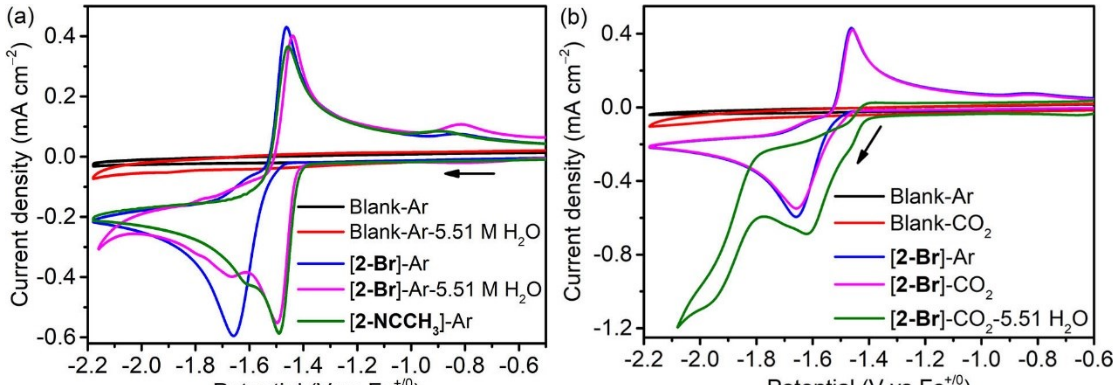

Figure S21. *(a) CVs of [**2**–**Br**] (1 mM) in anhydrous CH_3CN with TBAP (0.1 M) as electrolyte under Ar (blue), Ar with 5.51 M H2O added (purple) and CV of and [**2**–**MeCN**](OTf) (1 mM) in anhydrous CH_3CN with TBAP (0.1 M) as electrolyte under Ar (green); (b) CVs of [**2**–**Br**] (1 mM) in anhydrous CH_3CN with TBAP (0.1 M) as electrolyte under CO_2 (purple), CO2 with 5.51 M H2O added (green).*

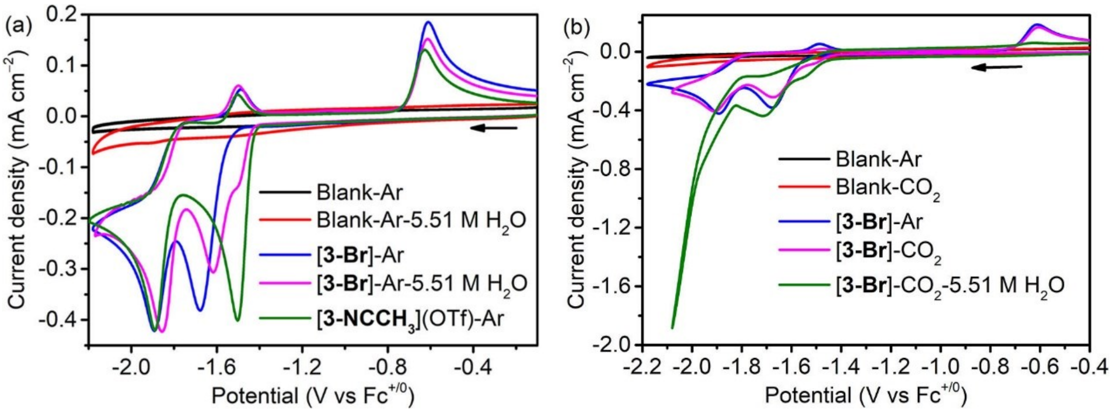

Figure S22. (a) CVs of [**3**–**Br**] (1 mM) in anhydrous CH_3CN with TBAP (0.1 M) as electrolyte under Ar (blue), Ar with 5.51 M H_2O added (purple) and CV of and [**3**–**MeCN**](OTf) (1 mM) in anhydrous CH_3CN with TBAP (0.1 M) as electrolyte under Ar (green); (b) CVs of [**3**–**Br**] (1 mM) in anhydrous CH_3CN with TBAP (0.1 M) as electrolyte under CO_2 (purple), CO_2 with 5.51 M H_2O added (green).

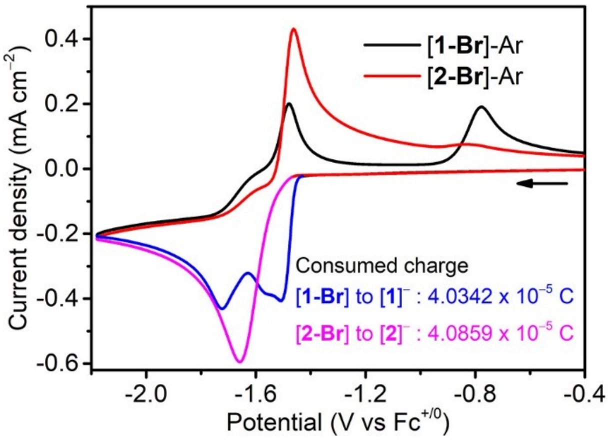

Figure S23. CVs of [**1**–**Br**] (1 mM) and [**2**–**Br**] (1 mM) in anhydrous CH_3CN with TBAP (0.1 M) as electrolyte under an inter atmosphere (Ar) with the peak integration corresponding to the consumed charges. Due to the steric influence of the amide –NMe_2 group, complex [**2**–**Br**] under dry conditions displayed a similar electrochemical property to complex [Mn(mesbpy)$(CO)_3$Br][1] which exhibited a single, two-electron reduction wave (Figure S24). As shown in Figure S23, the consumed charge of the reduction wave at –1.61 V (4.0859 x 10^{-5} C) for complex [**2**–**Br**] is similar with that of the sum of the first two one-electron processes of [**1**–**Br**] (4.0342 x 10^{-5} C). We thereby assigned the reduction wave as a two-electron reduction process.

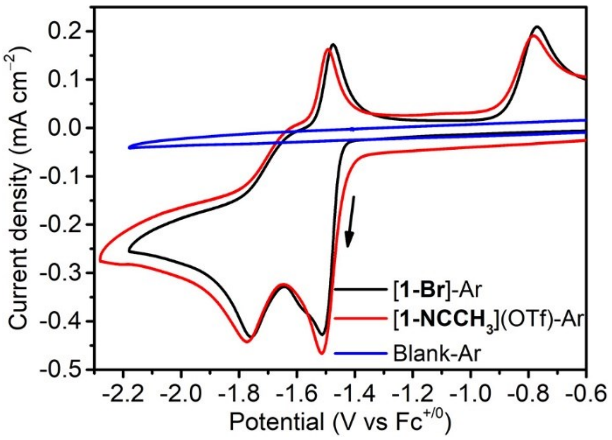

Figure S24. CVs of [**1**–**Br**] (1 mM) and [**1**–**MeCN**](OTf) (1 mM) in anhydrous CH_3CN with TBAP (0.1 M) as electrolyte under an inter atmosphere (Ar).

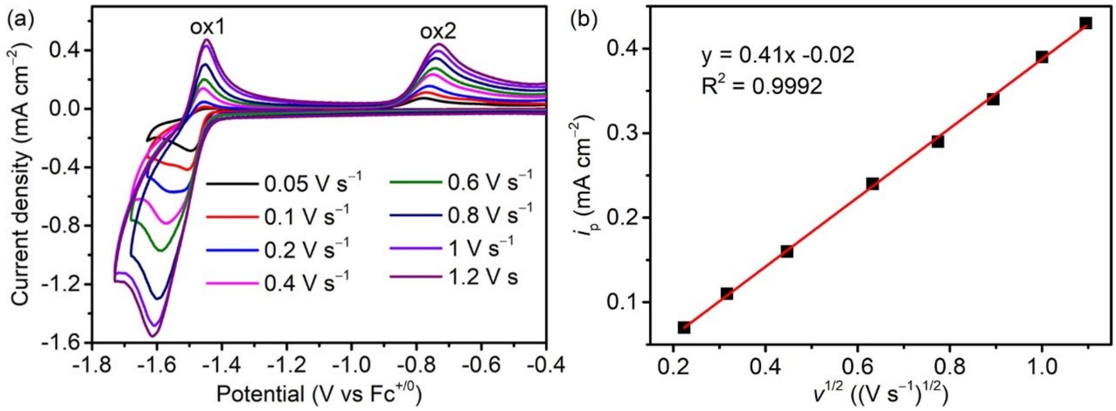

Figure S25. (a) CVs of [**1**–**Br**] (1 mM) in Ar-saturated CH_3CN with 0.1 M TBAP at different scan rate (0.05 – 1.2 V s^{-1}); (b) The linear plot of i_{ox2} versus $v^{1/2}$ for CVs of [**1**–**Br**].

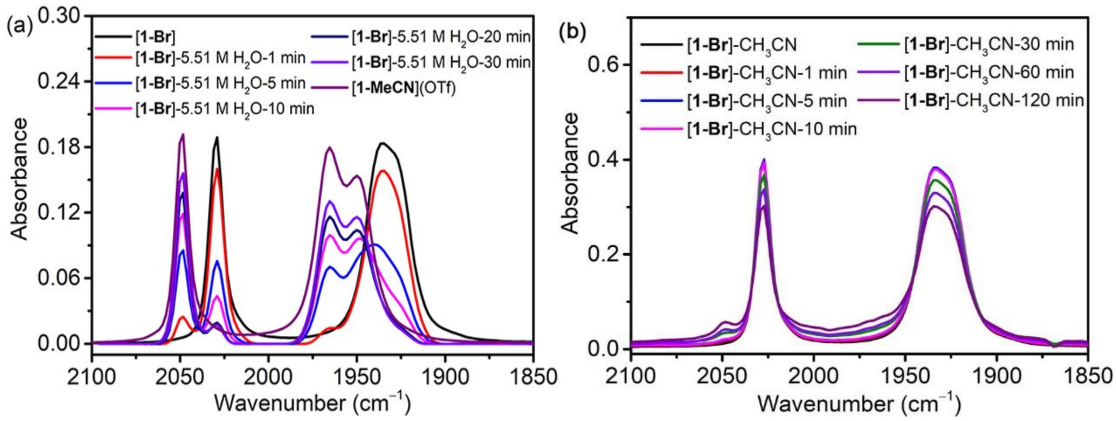

Figure S26. *FTIR spectral change versus time plot of complex [**1**–**Br**] (1 mM) in (a) CH_3CN solution with 5.51 M H_2O and (b) CH_3CN solution.*

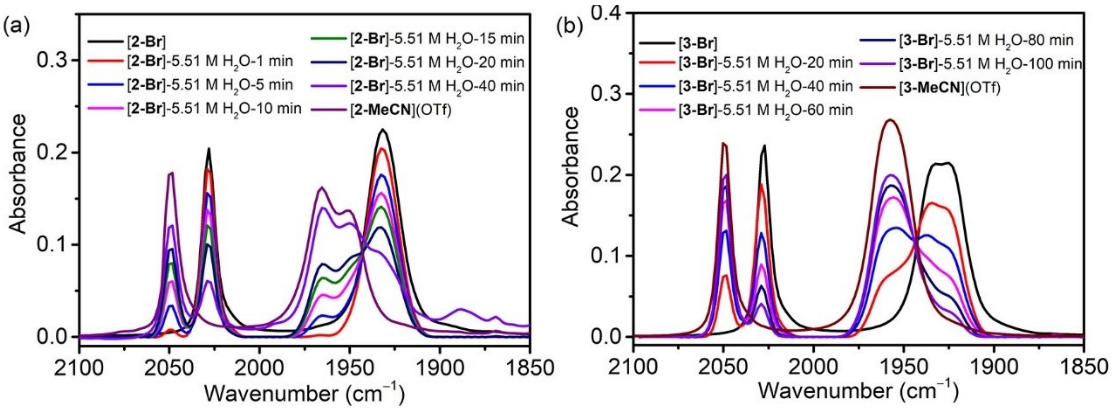

Figure S27. (a) FTIR spectral change versus time plot of complex [**2**–**Br**] (1 mM) in CH_3CN solution with 5.51 M H_2O and FTIR spectrum of complex [**2**–**MeCN**](OTf) in CH_3CN solution; (b) FTIR spectral change versus time plot of complex [**3**–**Br**] (1 mM) in CH_3CN solution with 5.51 M H_2O and FTIR spectrum of complex [**3**–**MeCN**](OTf) in CH_3CN solution.

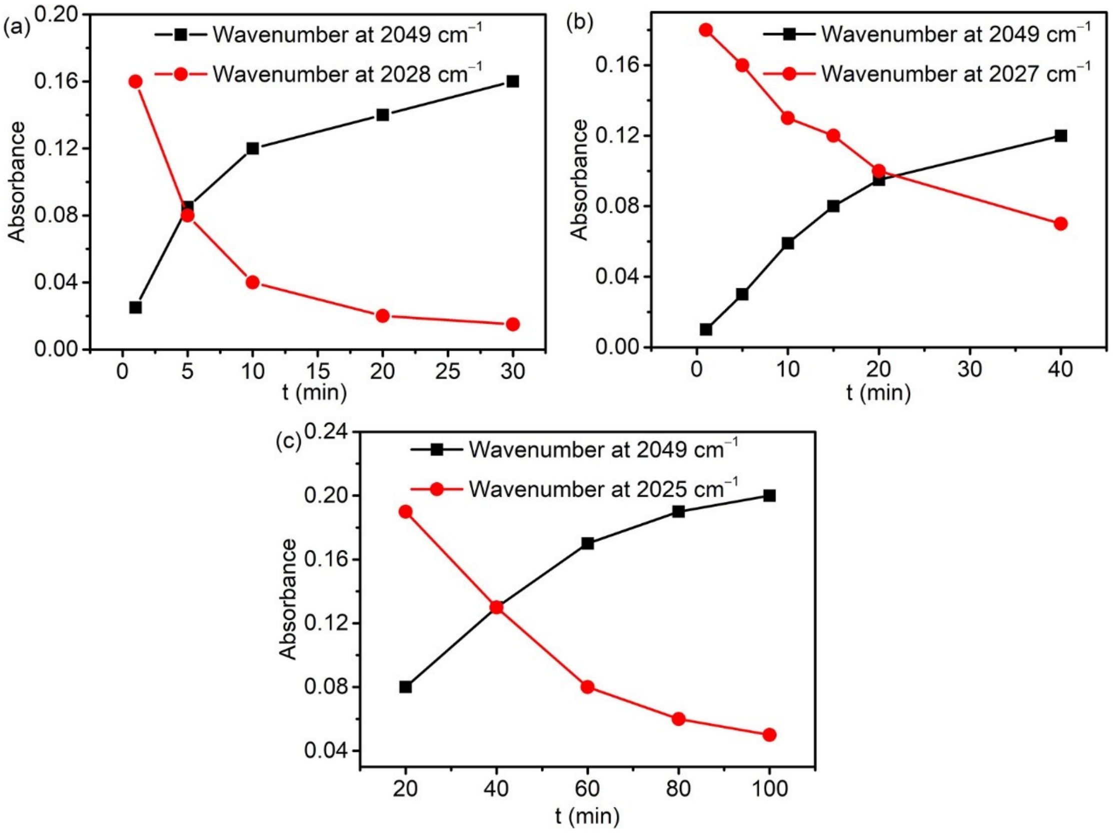

Figure S28. The plots of v_{CO} absorbance as a function of time for (a) [**1**–**Br**] (1 mM) at 2049 and 2028 cm^{-1}, (b) [**2**–**Br**] (1 mM) at 2049 and 2027 cm^{-1} and (c) [**3**–**Br**] (1 mM) at 2049 and 2025 cm^{-1}. Reaction conditions: CH_3CN solution with 5.51 M H_2O.

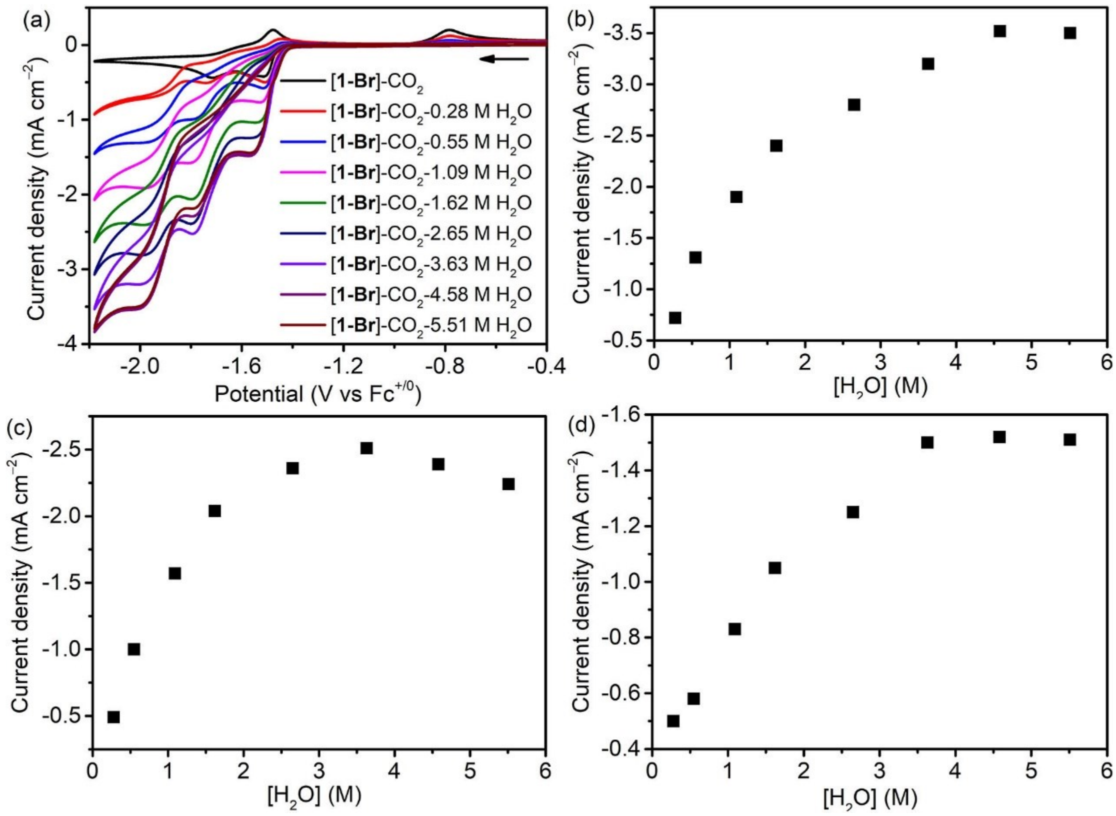

Figure S29. (a) CVs of the [**1**–**Br**] (1 mM) in CH_3CN with TBAP (0.1 M) as electrolyte under saturated CO_2 with the addition of H_2O (0.28–5.51 M H_2O); The plots of the catalytic current as a function of the water concentration for the (b) third, (c) second and (d) first catalytic waves.

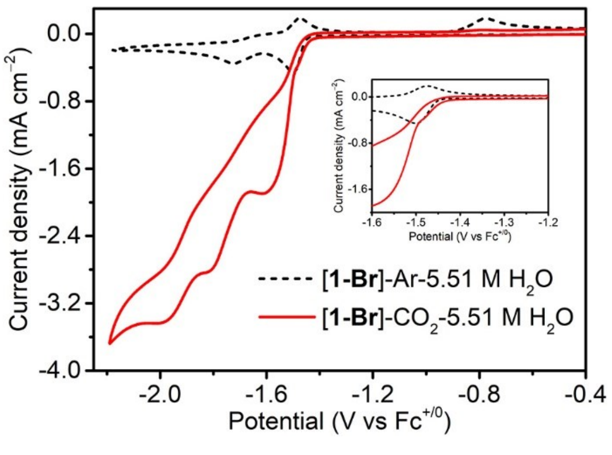

Figure S30. CVs of [**1**–**Br**] (1 mM) with 5.51 M H_2O added in anhydrous CH_3CN with TBAP (0.1 M) as electrolyte under Ar (black), CO_2 (red) atmosphere.

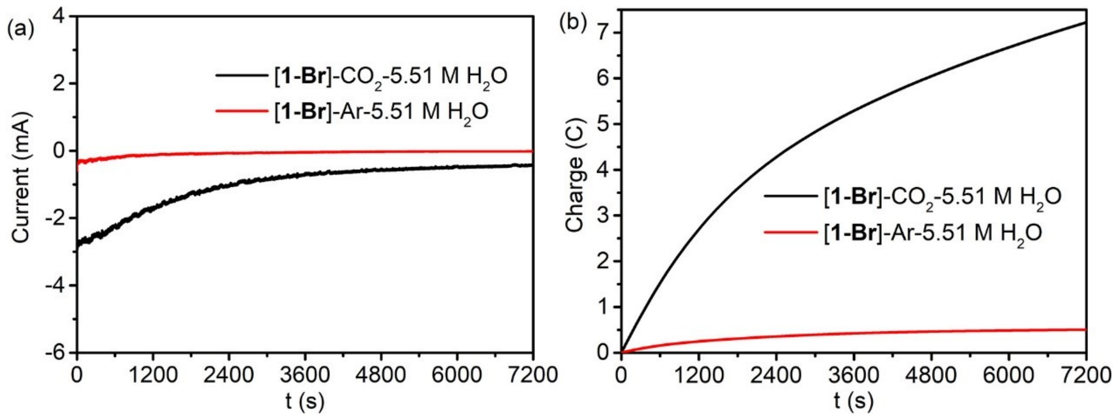

Figure S31. (a) Current vs time plots for the CPE and (b) charge passed during CPE of [**1**–**Br**] (1 mM) in 0.1 M TBAP/CH_3CN with 5.51 M H_2O at E_{app} = −1.55 V under CO_2-saturated atmosphere (black) and under Ar-saturated atmosphere (red).

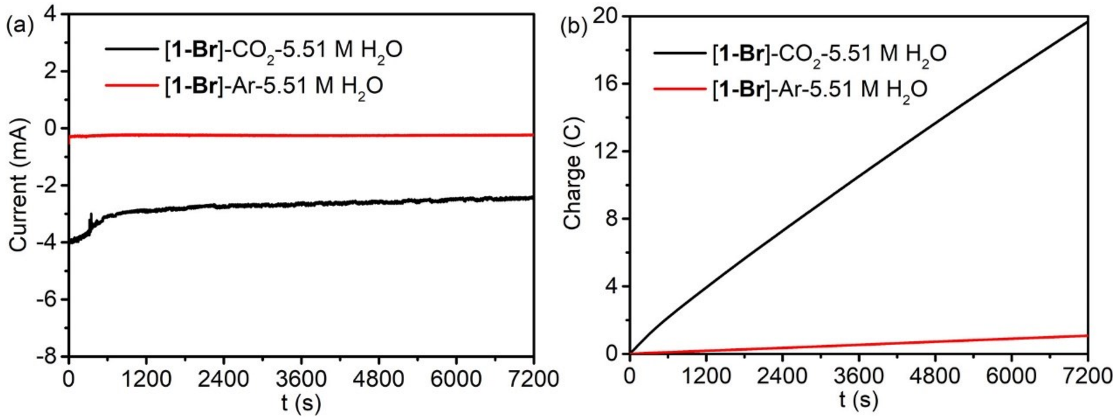

Figure S32. Current vs time plots for the CPE and (b) charge passed during CPE of [**1**–**Br**] (1 mM) in 0.1 M TBAP/CH_3CN with 5.51 M H_2O at E_{app} = −1.85 V under CO_2-saturated atmosphere (black) and under Ar-saturated atmosphere (red).

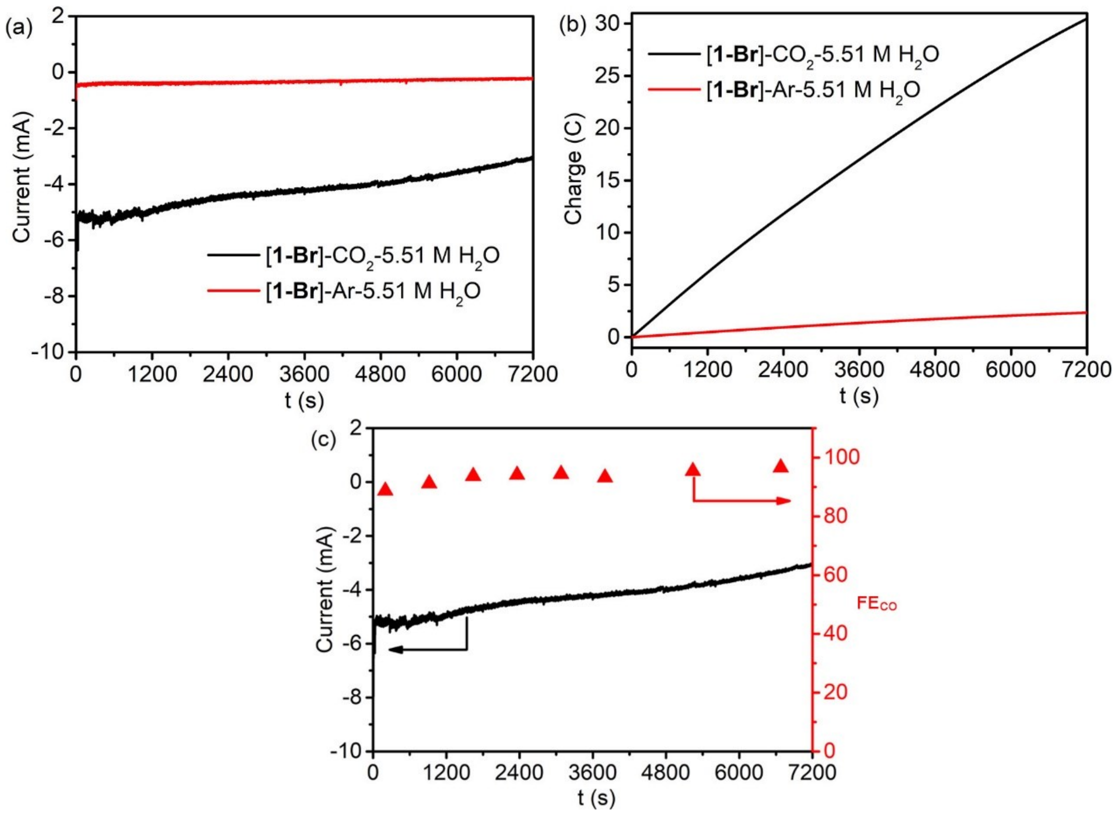

Figure S33. (a) Current vs time plots for the CPE and (b) charge passed during CPE of [**1**–**Br**] (1 mM) in 0.1 M TBAP/CH_3CN under CO_2 with 5.51 M H_2O at E_{app} = –2.05 V; (c) Faradaic efficiency for CO production over electrolysis time during CPE of [**1**–**Br**] (1 mM) in 0.1 M TBAP/CH_3CN under CO_2 with 5.51 M H_2O at E_{app} = –2.05 V.

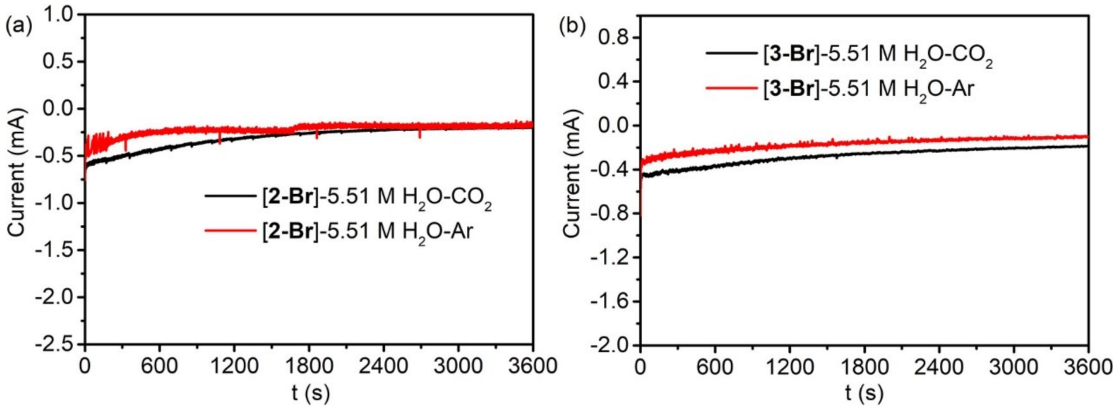

Figure S34. Current vs time plots for the CPE of complexes (a) [**2**–**Br**] and (b) [**3**–**Br**] (1 mM) in 0.1 M TBAP/CH_3CN with 5.51 M H_2O at E_{app} = −1.85 V under CO_2-saturated atmosphere (black) and under Ar-saturated atmosphere (red).

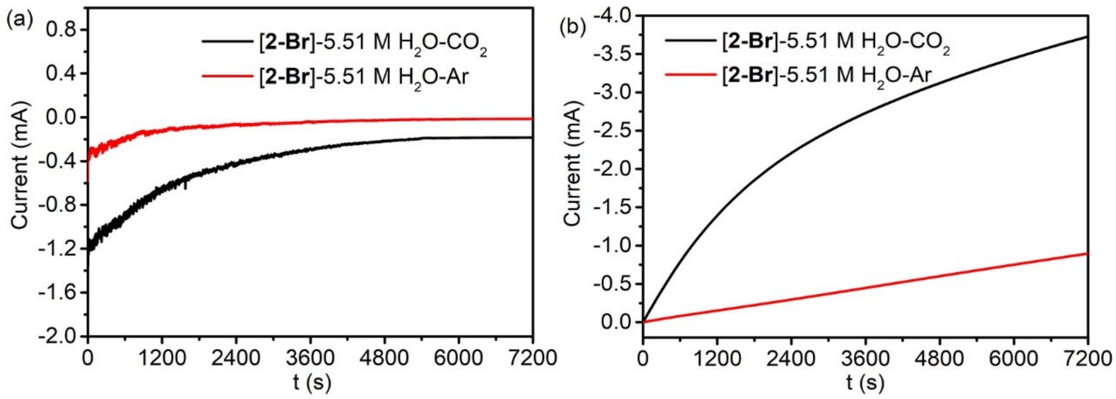

Figure S35. (a) Current vs time plots for the CPE and (b) charge passed during CPE of [**2**–**Br**] (1 mM) in 0.1 M TBAP/CH_3CN with 5.51 M H_2O at E_{app} = −2.05 V under CO_2-saturated atmosphere (black) and under Ar-saturated atmosphere (red).

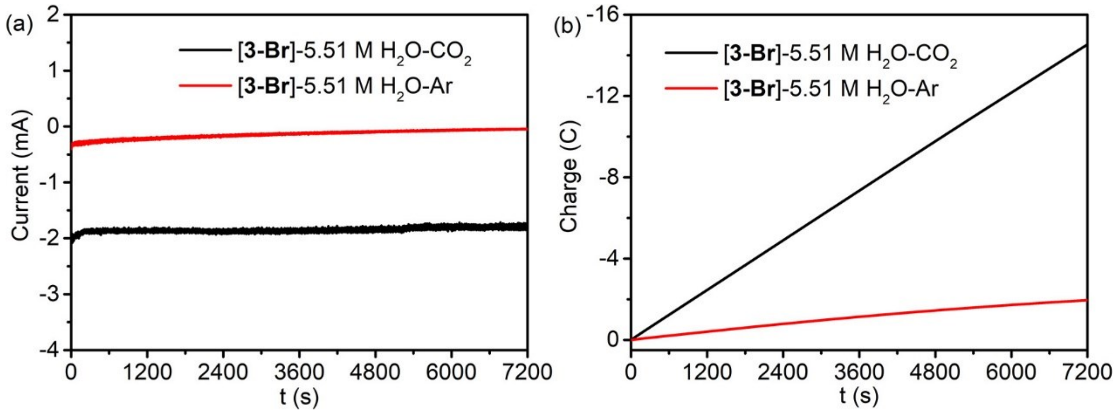

Figure S36. (a) Current vs time plots for the CPE and (b) charge passed during CPE of [**3**–**Br**] (1 mM) in 0.1 M TBAP/CH_3CN with 5.51 M H_2O at E_{app} = −2.05 V under CO_2-saturated atmosphere (black) and under Ar-saturated atmosphere (red).

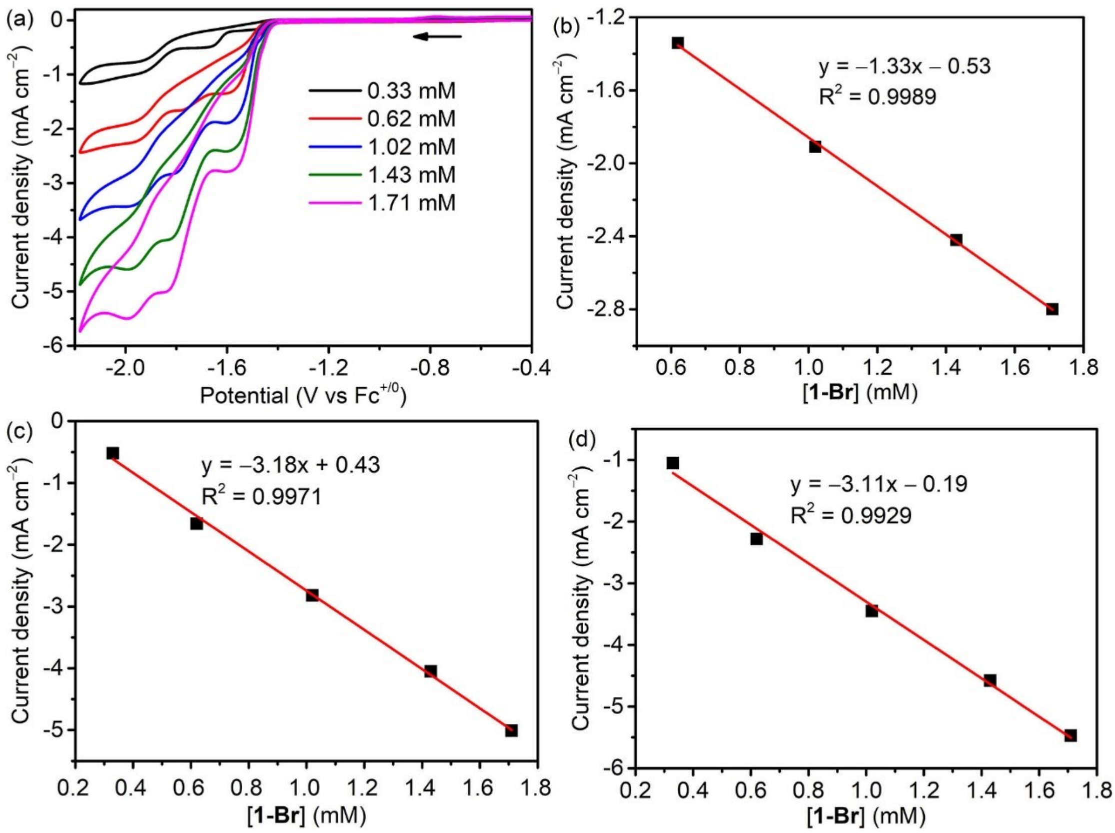

Figure S37. (a) CVs of complex [**1**–**Br**] at various concentrations (0.33–1.71 mM) in CO_2-saturated CH_3CN with 0.1 M TBAP as electrolyte at scan rate 100 mV s^{-1}; The linear plot of i_{cat} versus catalyst concentration of (b) first, (c) second and (d) third catalytic waves for CVs of [**1**–**Br**].

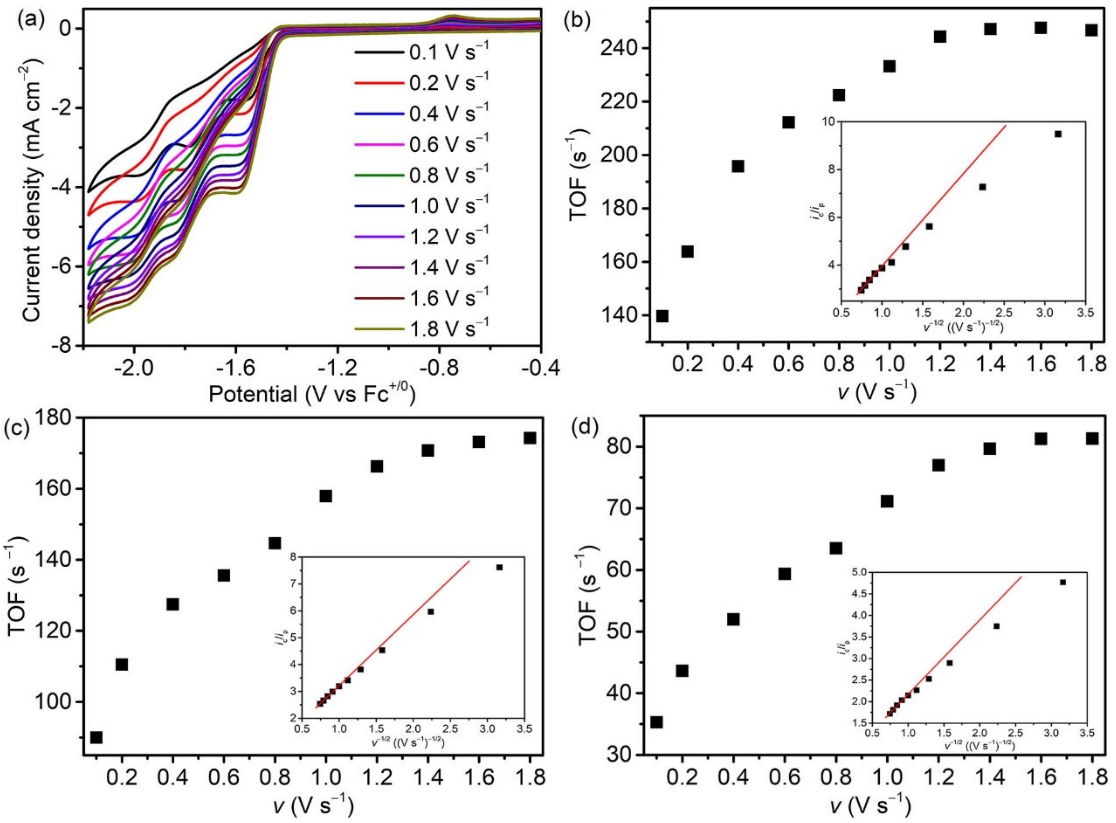

Figure S38. *(a) CVs of complex [**1**–**Br**] (1 mM) in CO_2-saturated CH_3CN solution with 0.1 M TBAP and 5.51 M H_2O as electrolyte at different scan rates (0.1 to 1.8 V s^{-1}); The plots of TOF versus scan rate for (b) third catalytic wave, (c) second catalytic wave and (d) first catalytic wave, with an inset of i_c/i_p versus inverse square root of the scan rate, highlighting that steady-state conditions are accomplished at high scan rates (1.4–1.8 V s^{-1}).*

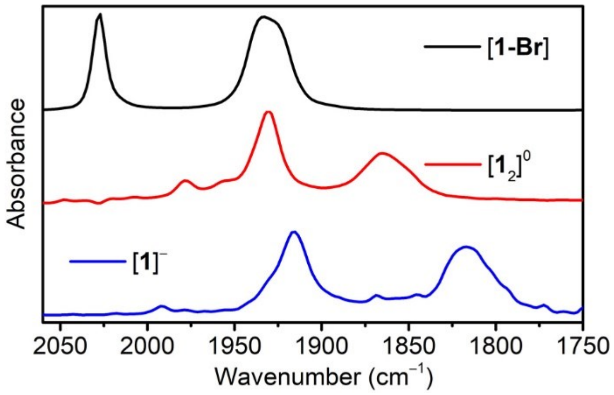

Figure S39. FTIR-SEC of [**1**–**Br**] (5 mM) in CH_3CN solution (0.05 M TBAP, 5.51 M H_2O) under Ar: resting state (black), singly reduced species (red), and doubly reduced species (blue).

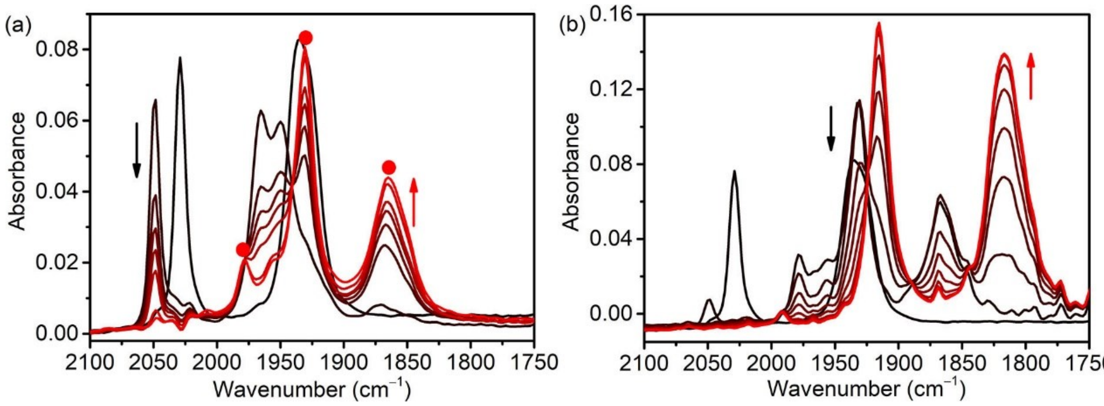

Figure S40. FTIR-SEC changes observed during the reaction of [**1**–**Br**] (5 mM) in CH_3CN solution (0.05 M TBAP) with 5.51 M H_2O under Ar at applied potential (a) −1.55 V and (b) −1.75 V. Black and red curves describe the starting and the final spectra, respectively.

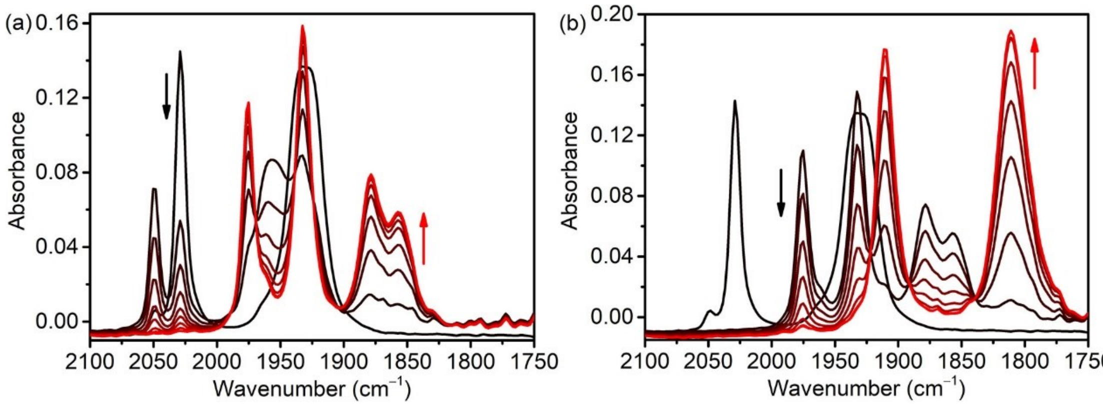

Figure S41. FTIR-SEC changes observed during the reaction of [**3**–**Br**] (5 mM) in CH_3CN solution (0.05 M TBAP) with 5.51 M H_2O under Ar at applied potential (a) −1.5 V and (b) −1.85 V. Black and red curves describe the starting and the final spectra, respectively.

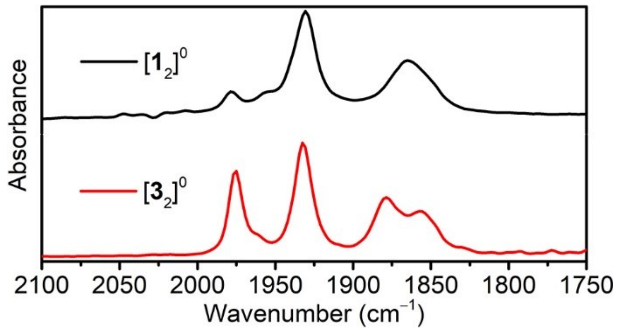

Figure S42. The v_{CO} stretching bands of FTIR-SEC spectra for complex [$\mathbf{1}_2$]^0 and [$\mathbf{3}_2$]^0 with 5.51 M H_2O added in CH_3CN with TBAP (0.1 M) as electrolyte under Ar.

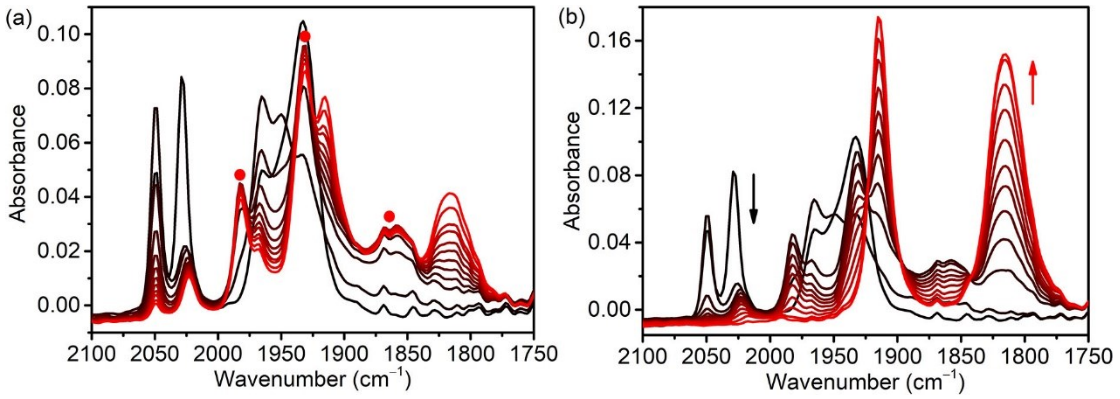

Figure S43. FTIR-SEC changes observed during the reaction of [**2**–**Br**] (5 mM) in CH_3CN solution (0.05 M TBAP) with 5.51 M H_2O under Ar at applied potential (a) −1.45 V and (b) −1.6 V. Black and red curves describe the starting and the final spectra, respectively.

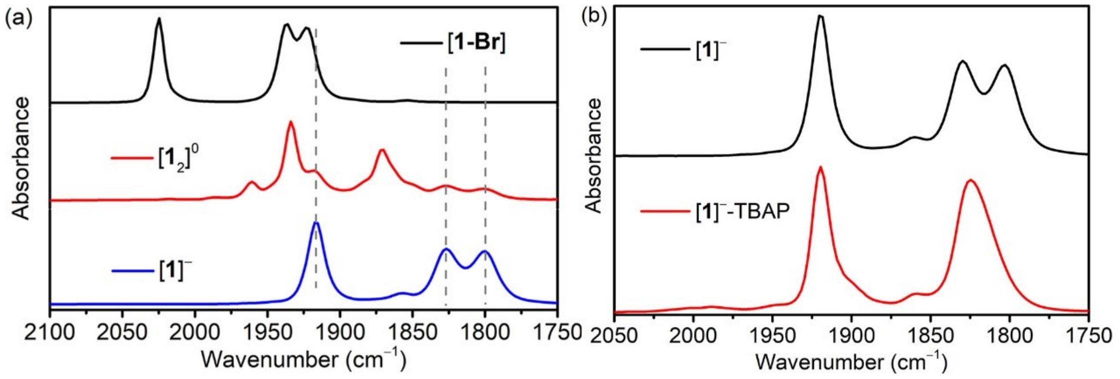

Figure S44. (a) FTIR spectra of [**1**–**Br**] (black) and its singly (red) and doubly reduced species (blue) prepared via chemical reduction with KC_8 in THF solutions; (b) The FTIR spectra of [**1**]$^-$ generated in the chemical reduction experiment without (black) and with (red) TBAP in THF solution.

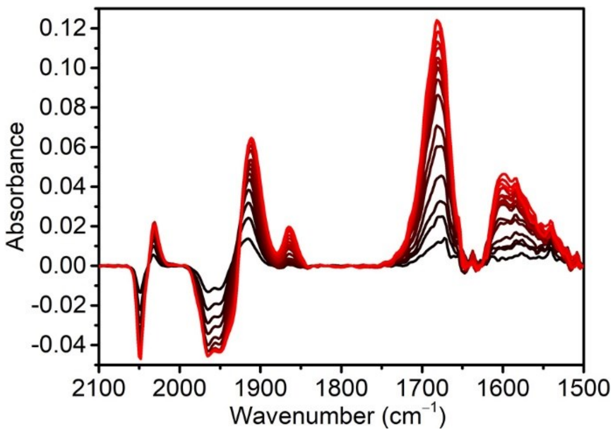

Figure S45. Differential FTIR-SEC spectra of [**1**–**Br**] (5 mM) in CO_2-saturated CH_3CN solution (0.05 M TBAP, 5.51 M H_2O) at the applied potential −1.75 V. Black and red curves describe the starting and the final spectra, respectively.

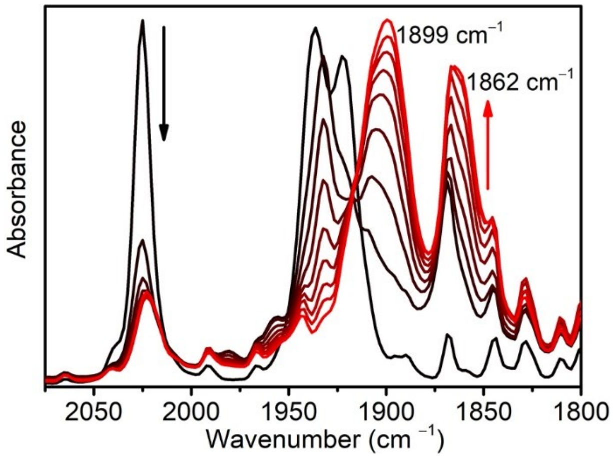

Figure S46. FTIR-SEC changes observed during the reaction (applied potential −1.75 V) of [**1**–**Br**] (5 mM) in THF solution (0.05 M TBAP, 5.51 M H_2O) under CO_2. Black and red curves describe the starting and the final spectra, respectively.

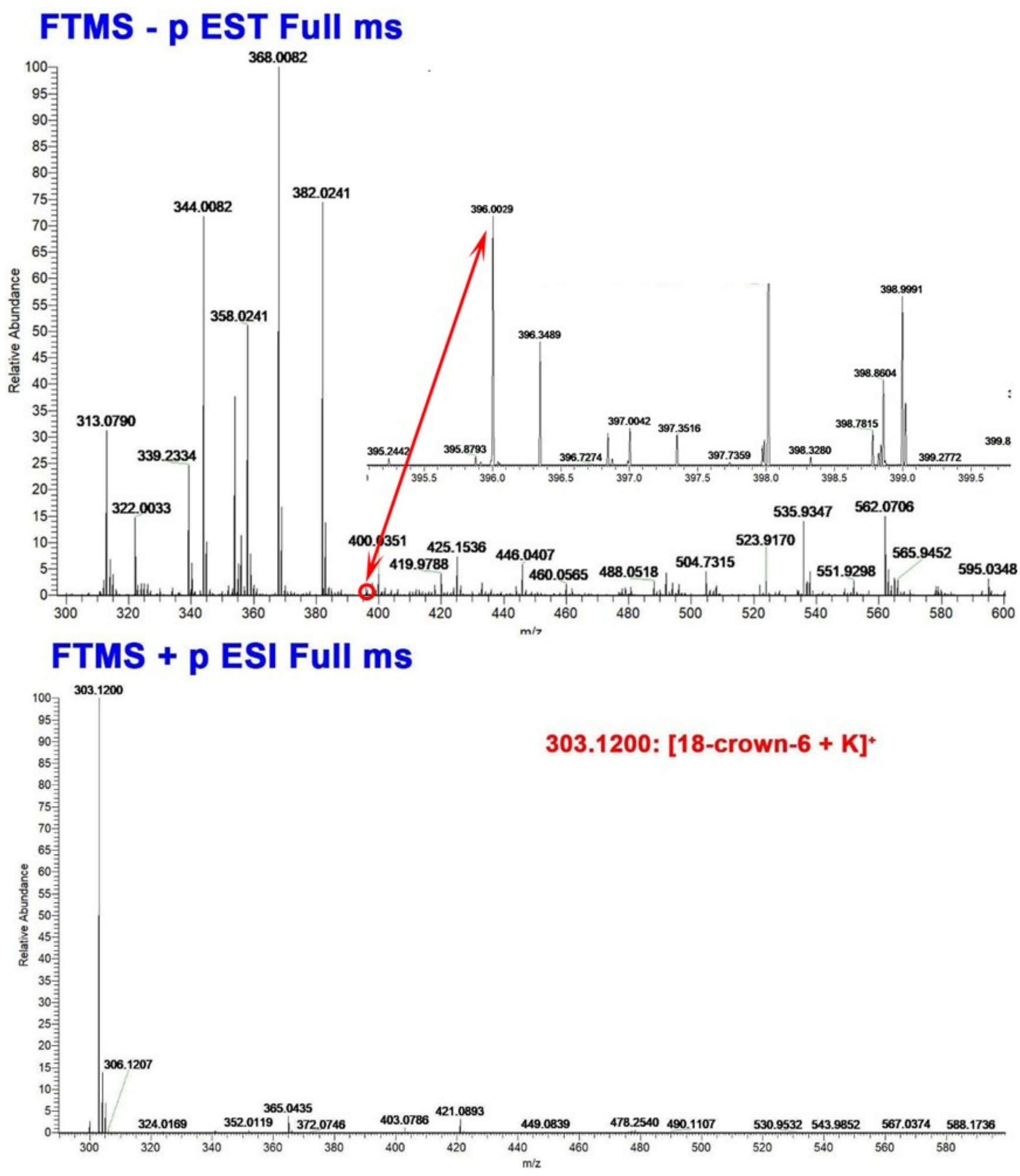

Figure S47. The HR-MS data of two-electrons reduced species [**1**]$^{-}$ mixed with CO_2-containing THF solution. Inset: Partial spectrum with mass ratio from 395 to 400.

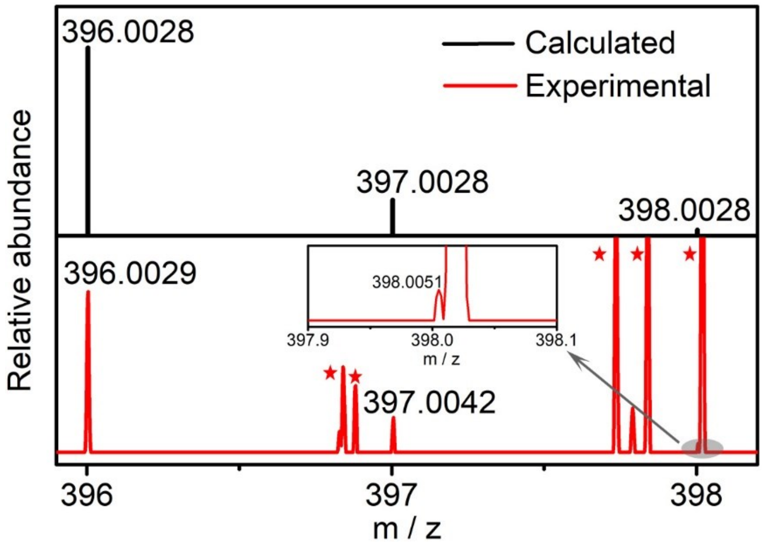

Figure S48. Experimentally observed (red) and calculated (black) spectra of (a) [**1** + $\mathbf{CO_2}$]$^-$ after mixing a CO_2-containing solution with [**1**]$^-$. Note: (1) the red-star labelled signals represent other unknown species; (2) the eluent used for the HR-MS measurements contains methanol.

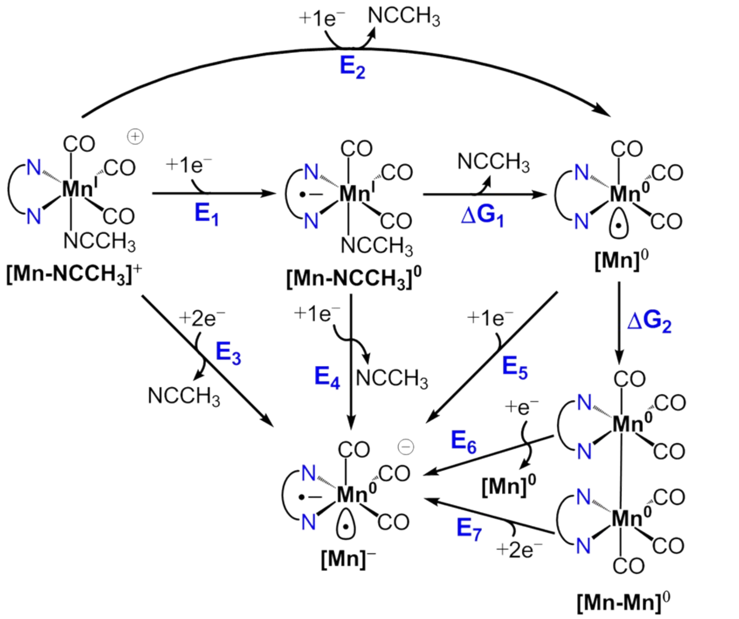

E_1	–1.73 V
ΔG_1	–4.7 kcal/mol
E_2	–1.52 V
E_3	–1.65 V
E_4	–1.58 V
E_5	–1.78 V
ΔG_2	–12.2 kcal/mol
E_6	–2.27
E_7	–2.03 V

Figure S49. Catalyst activation and dimerization pathways for [*fac*-Mn(bpy-CONHMe)(CO)$_3$NCCH$_3$]$^+$ ([**Mn–NCCH$_3$**]$^+$ or [**1–NCCH$_3$**]$^+$).

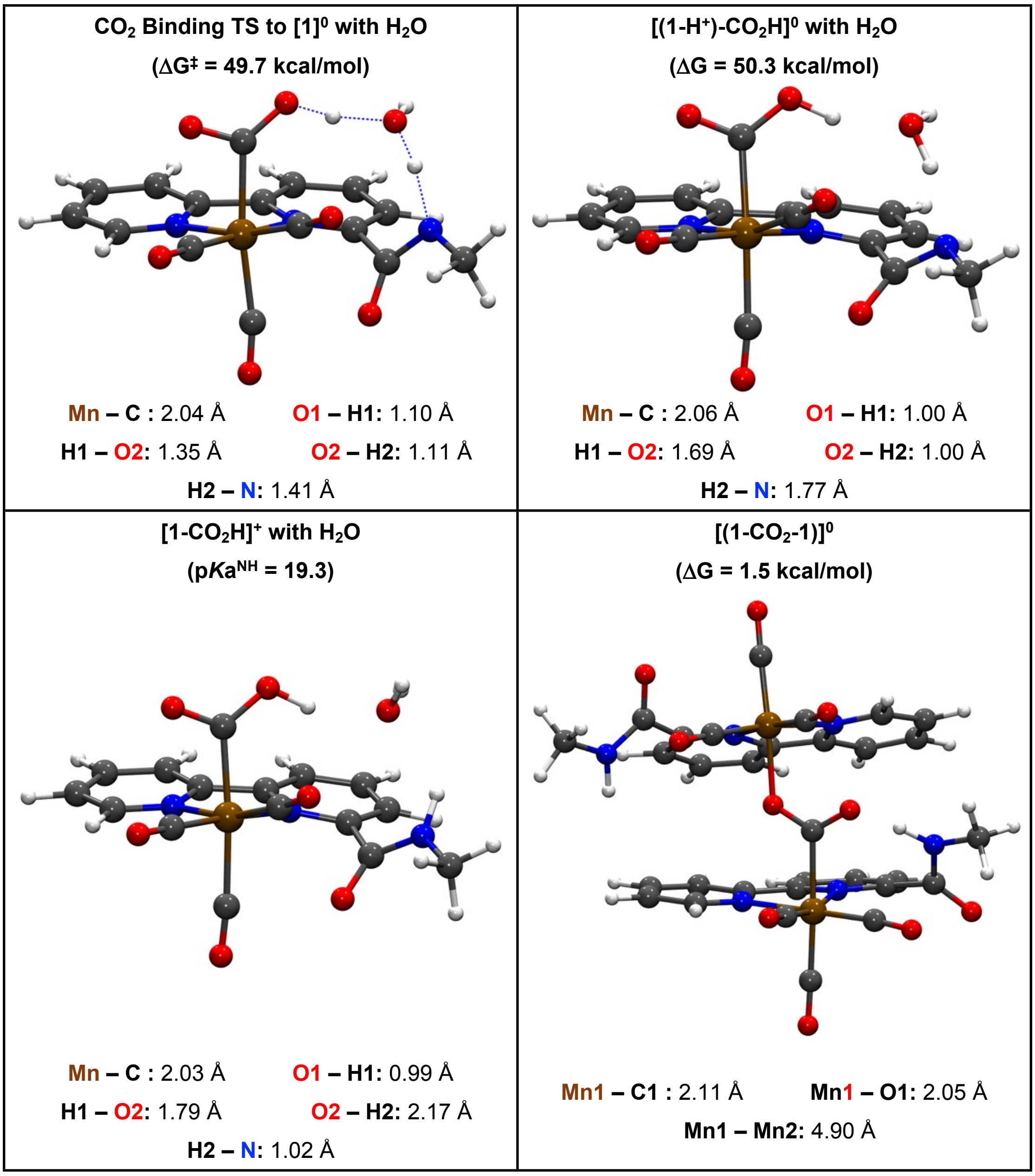

Figure S50. Optimized **(a)** transition state structure for CO_2 binding to [1]^0, **(b)** structure for Mn^{II}-COOH species with deprotonated ligand (**[(1-H$^+$)-CO$_2$H]^0**) and **(c)** structure for Mn^{II}-COOH (**[1-CO$_2$H]$^+$**) with and a water molecule. ΔG‡ and ΔG are in units of kcal/mol with respect to separated reactants **[1]^0**, H_2O and CO_2.

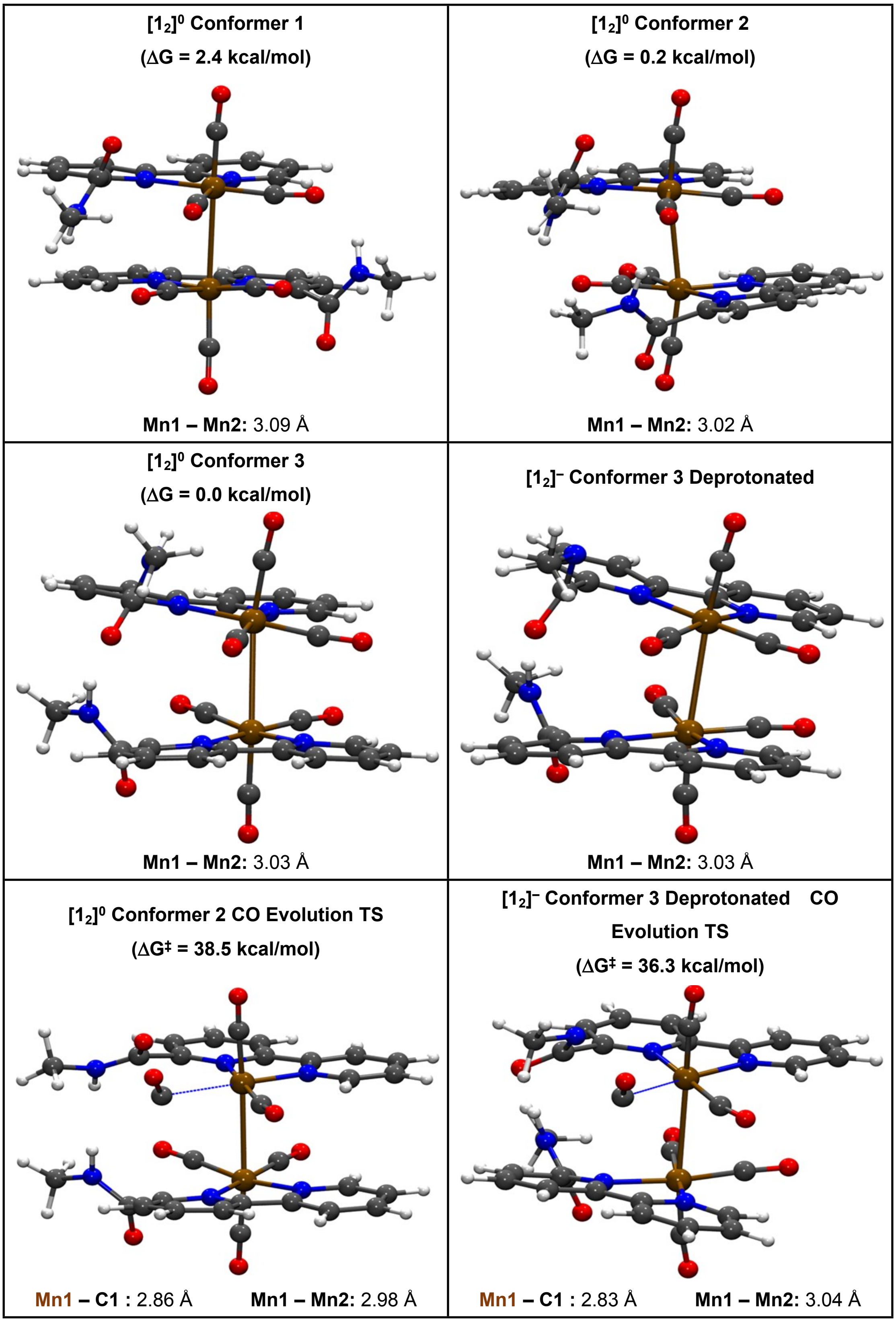

Figure S51. Optimized structures for **$[1_2]^0$ (a-c), (d)** deprotonated form of **$[1_2]^0$**, transition states for CO evolution from **(e) $[1_2]^0$** conformer 1 and **(f)** deprotonated form of **$[1_2]^0$** conformer 3. $\Delta G^‡$ and ΔG are in units of kcal/mol with respect to most stable **$[1]^0$** conformer 3 except for last TS for which the reference is deprotonated **$[1_2]^-$** conformer 3.

$[1_2]^0$–CO Conformer 1

(ΔG = 22.2 kcal/mol)

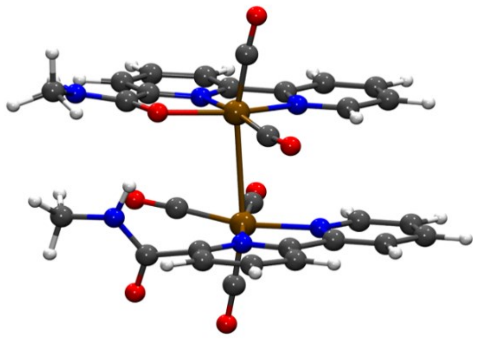

Mn1 – Mn2: 3.07 Å

$[1_2]^0$–CO + CO_2 + H_2O Conformer 1

(ΔG = 63.6 kcal/mol)

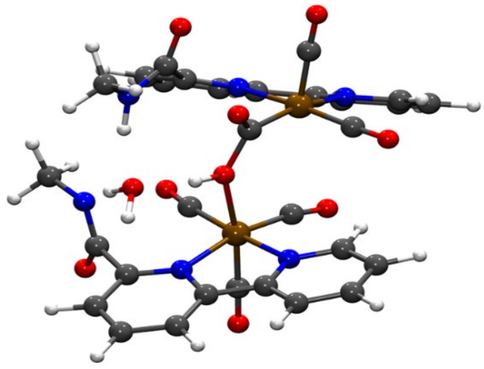

Mn1 – C1 : 1.97 Å **Mn1 – O1:** 2.13 Å

Mn1 – Mn2: 3.87 Å

$[1_2]^0$–CO + CO_2 + H_2O Conformer 2

(ΔG = 55.3 kcal/mol)

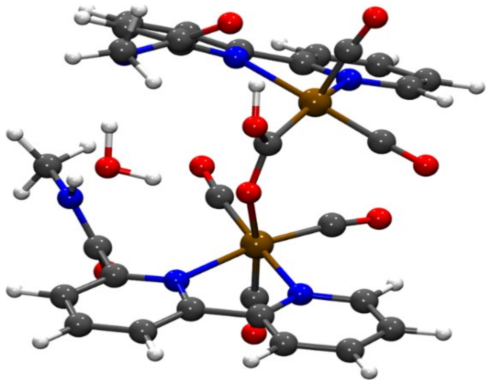

Mn1 – C1 : 1.96 Å **Mn1 – O1:** 2.07 Å

Mn1 – Mn2: 3.68 Å

$[1_2]^0$–CO + CO_2 + H_2O Conformer 3

(ΔG = 58.1 kcal/mol)

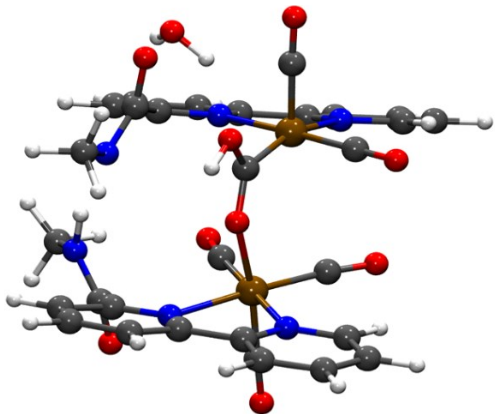

Mn1 – C1 : 1.93 Å **Mn1 – O1:** 2.05 Å

Mn1 – Mn2: 3.75 Å

$[1_2]^0$–CO + CO_2 + H_2O Conformer 4

(ΔG = 56.8 kcal/mol)

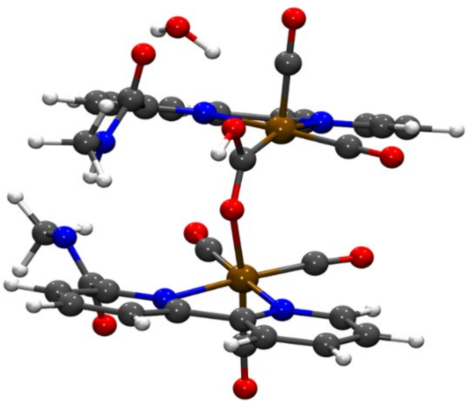

Mn1 – C1 : 1.94 Å **Mn1 – O1:** 2.06 Å

Mn1 – Mn2: 3.73 Å

$[1_2]^0$–CO + CO_2 + H_2O Conformer 5

(ΔG = 58.7 kcal/mol)

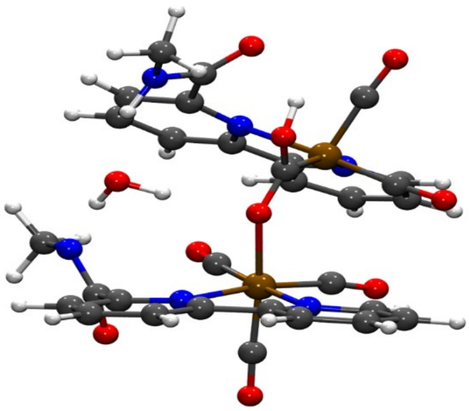

Mn1 – C1 : 1.95 Å **Mn1 – O1:** 2.10 Å

Mn1 – Mn2: 3.71 Å

Figure S52. Optimized structures for **(a) $[1_2]^0$-CO** where one of the CO molecules has dissociated**, (b-f)** different conformers of species fromed upon addition of CO_2 and H_2O to **$[1_2]^0$-CO**. ΔGs are in units of kcal/mol with respect to separated reactants **$[1_2]^0$**, H_2O, CO_2 and CO.

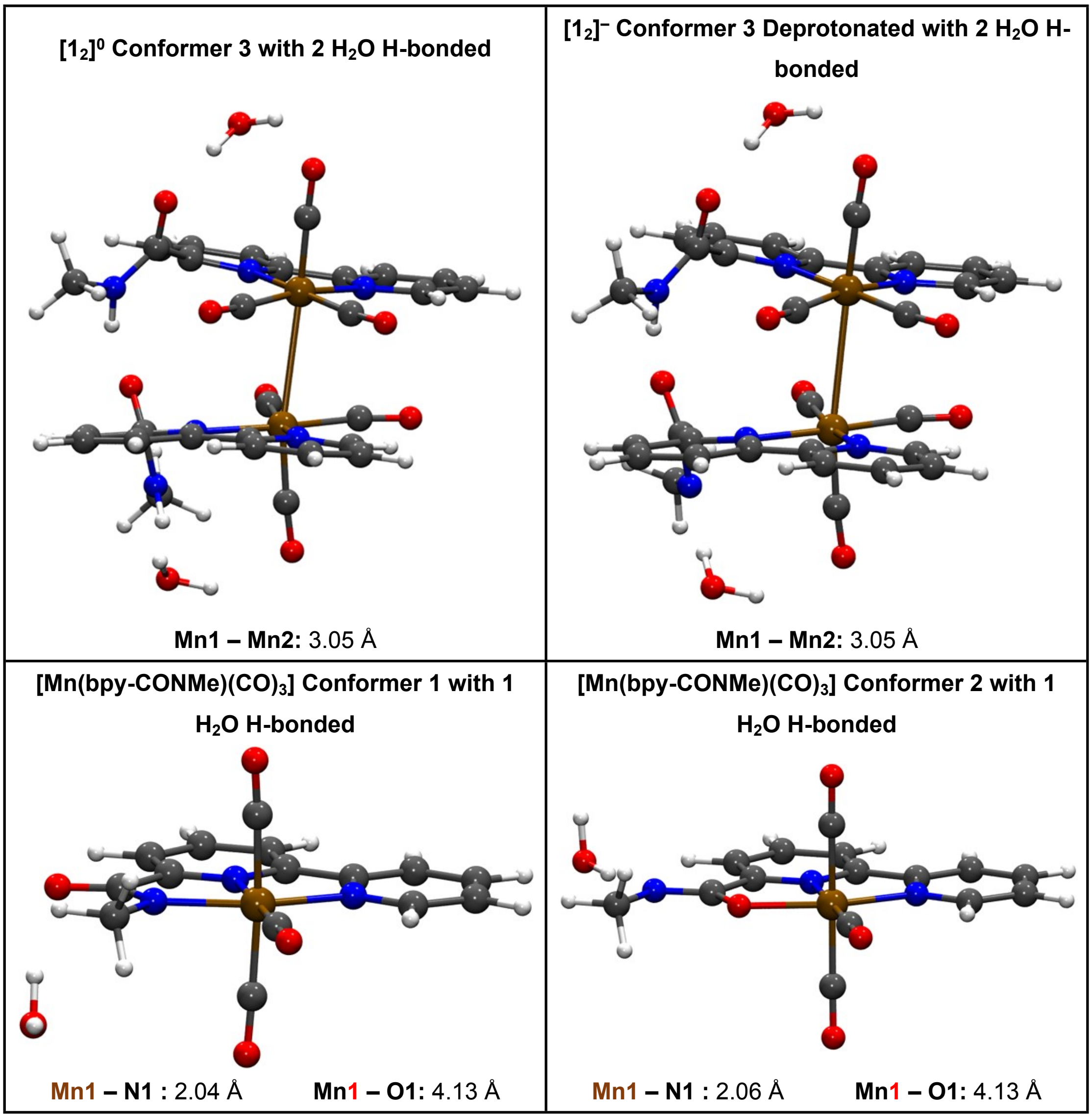

Figure S53. Optimized structures for **(a)** **$[1_2]^0$** conformer 3 with 2 H_2O H-bonded, **(b)** $[1_2]^-$ Conformer 3 Deprotonated with 2 H_2O, **(c)** [Mn(bpy-CONMe)$(CO)_3$] Conformer 1 with 1 H_2O H-bonded and **(d)** [Mn(bpy-CONMe)$(CO)_3$] conformer 2 with 1 H_2O H-bonded.

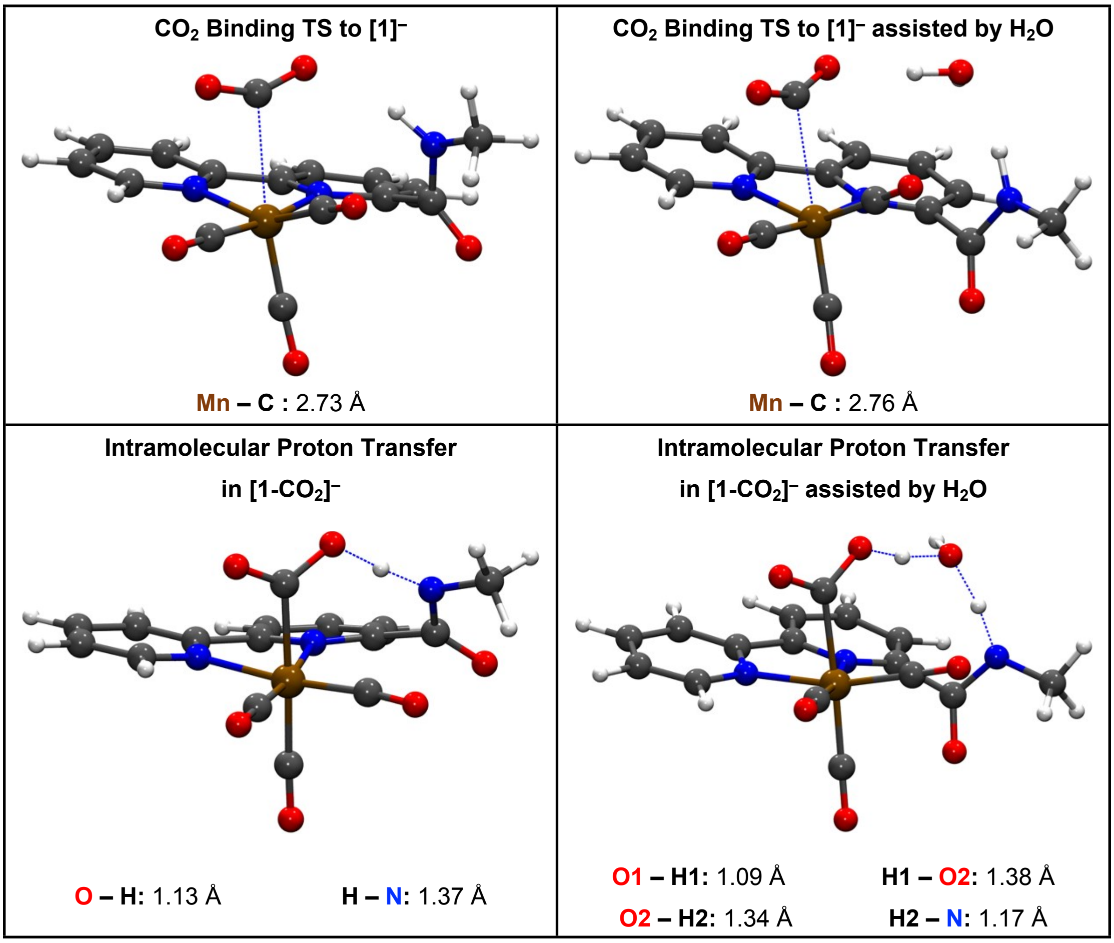

Figure S54. Optimized transition state structures for CO_2 binding to [**1**]$^-$ (top) and intramolecular proton transfer in [**1-CO$_2$**]$^-$ (bottom) with and without assistance of a water molecule.

**[1-CO_2H]^0 C–OH bond clevage TS**<br>**with 1 H_2O Assisted by Amide Group**<br>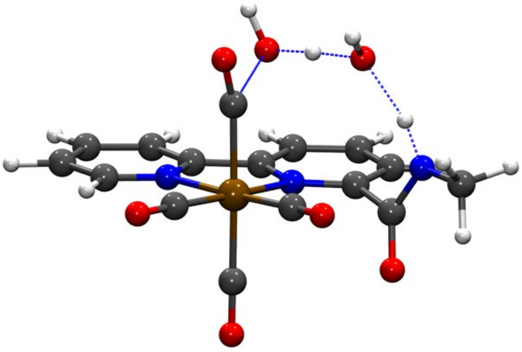<br>C – O1: 2.34 Å O1 – H1: 1.10 Å<br>H1 – O2: 1.36 Å O2 – H2: 1.56 Å<br>H2 – N: 1.08 Å	**[1-CO_2H]$^–$ C–OH bond clevage TS**<br>**with 1 H_2O Assisted by Amide Group**<br>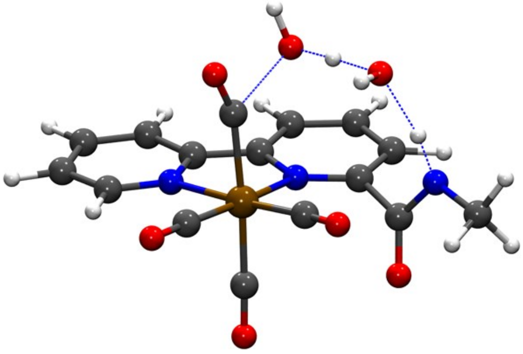<br>C – O1: 2.29 Å O1 – H1: 1.11 Å<br>H1 – O2: 1.35 Å O2 – H2: 1.54 Å<br>H2 – N: 1.09 Å
**[1-CO_2H]^0 C–OH bond clevage TS**<br>**with 1 H_2O**<br>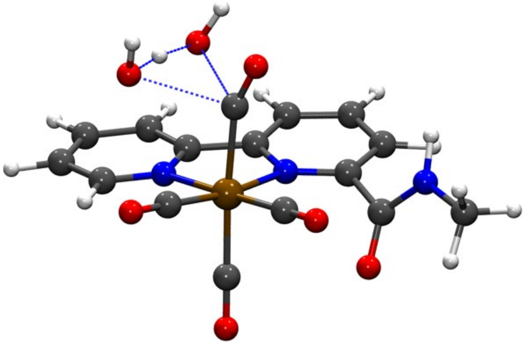<br>C – O1: 2.38 Å O1 – H1: 1.22 Å<br>H1 – O2: 1.20 Å O2 – C: 2.34 Å	**[1-CO_2H]$^–$ C–OH bond clevage TS**<br>**with 1 H_2O**<br>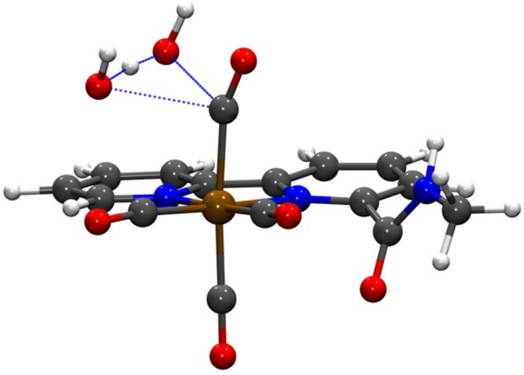<br>C – O1: 2.48 Å O1 – H1: 1.22 Å<br>H1 – O2: 1.20 Å O2 – C: 2.47 Å
**[1-CO_2H]^0 C–OH bond clevage TS**<br>**with 2 H_2O**<br>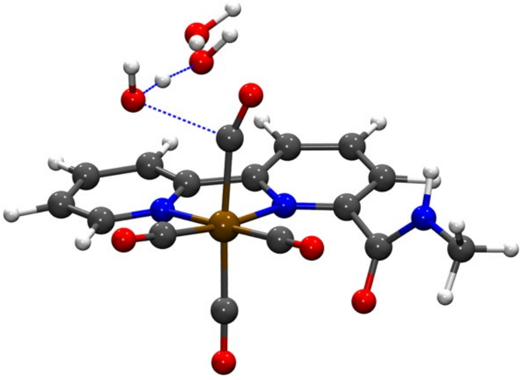<br>C – O1: 2.29 Å O1 – H1: 1.13 Å<br>H1 – O2: 1.31 Å O2 – C: 2.82 Å	**[1-CO_2H]$^–$ C–OH bond clevage TS**<br>**with 2 H_2O**<br>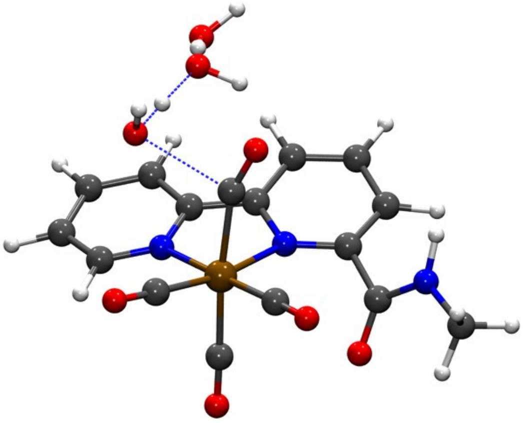<br>C – O1: 2.44 Å O1 – H1: 1.10 Å<br>H1 – O2: 1.38 Å O2 – C: 3.41 Å

Figure S55. Optimized transition state structures for C–OH bond cleavage for [**1-CO_2H**]^0 and [**1-CO_2H**]$^–$ species using H_2O as the weak Brønsted acid.

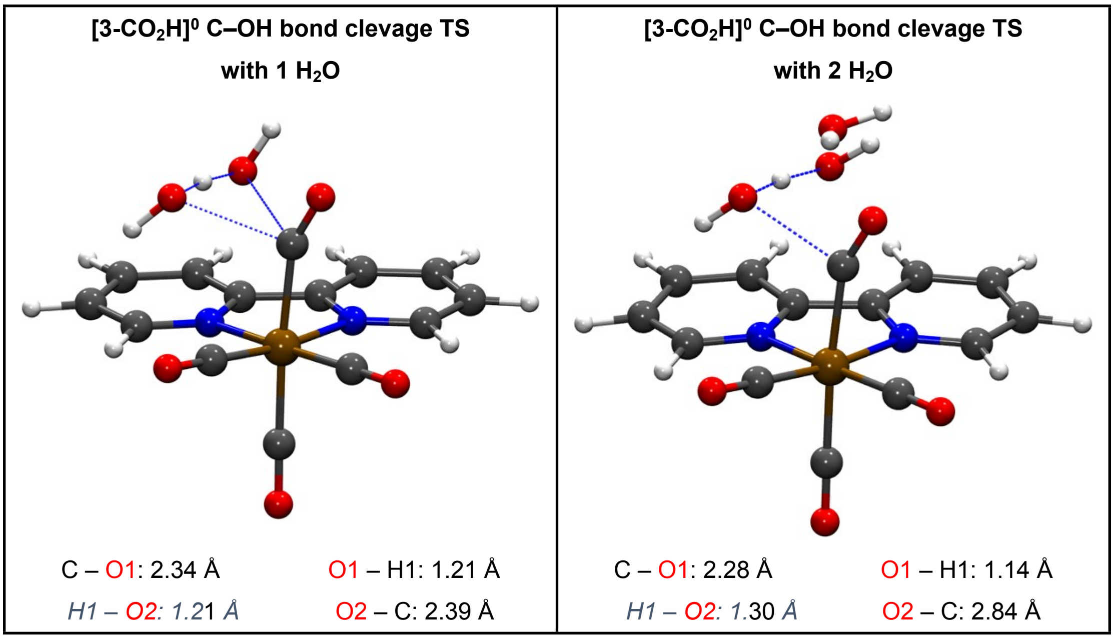

Figure S56. Optimized transition state structures for C–OH bond cleavage for $[Mn(bpy)(CO)_3CO_2H]^0$ (**[3-CO_2H]**^{0}) with 1 (left) and 2 (right) H_2O molecules as the weak Brønsted acid.

Table S1. Representative bond lengths [Å] for [**1**–**Br**].

	**[1–Br]**
Mn-C1	1.818
Mn-N1	2.032
N1-C2	1.345
C2-C3	1.382
C3-C4	1.380
C4-C5	1.388
C5-C6	1.391
C6-C7	1.480
C7-C8	1.385
C8-C9	1.381
C9-C10	1.389
C10-C11	1.383
C11-N2	1.351
Mn-N2	2.067
Mn-C13	1.808
Mn-C14	1.818
C1-O1	1.144
C13-O2	1.153
C14-O3	1.109

Table S2. Crystal data and structure refinement for [**1**–**Br**].

**Empirical formula**	**$C1_5H_{11}N_3O_4MnBr$**
Formula weight	430.93
Temperature	100 K
Wavelength	0.71073
Crystal system	Monoclinic
Space group	P 21/c
Unit cell dimensions	a = 10.650(4) Å α = 90º<br>b = 9.121(4) Å β = 100.266º<br>c = 16.456(14) γ = 90º
Volume	1572.9(11) Å^3
Density (calculated)	1.523 mg/m^3
Absorption coefficient	1.985
F(000)	856.0
Crystal size	0.42 x 0.38 x 0.34
Reflections collected	29067
Absorption correction	Muti-scan
Data / restraints / parameters	3618/0/219
Goodness-of-fit on F2	1.038
R indices (all data)	R1 = 0.0287 wR2 = 0.0709

Table S3. Representative bond lengths [Å] for [K(18-crown-6)]$^{+}$[**1**]$^{–}$.

	[K(18-crown-6)]$^{+}$[**1**]$^{–}$
Mn-C1	1.768
Mn-N1	1.973
N1-C2	1.370
C2-C3	1.363
C3-C4	1.426
C4-C5	1.340
C5-C6	1.406
C6-C7	1.401
C7-C8	1.417
C8-C9	1.354
C9-C10	1.395
C10-C11	1.372
C11-N2	1.391
Mn-N2	2.005
Mn-C13	1.749
Mn-C14	1.751
C1-O1	1.190
C13-O2	1.181
C14-O3	1.190

Table S4. Crystal data and structure refinement for $[K(18\text{-crown-6})]^+[\mathbf{1}]^-$.

Empirical formula	$C_{27}H_{35}N_3O_{10}KMn$
Formula weight	655.13
Temperature	100 K
Wavelength	0.71073
Crystal system	Monoclinic
Space group	P 21/c
Unit cell dimensions	a = 9.4091(12) Å α = 90º<br>b = 15.768(3) Å β = 94.907º<br>c = 25.766(5) γ = 90º
Volume	3808.7(11) $Å^3$
Density (calculated)	1.395 mg/m^3
Absorption coefficient	0.520
F(000)	1688
Crystal size	0.34 x 0.26 x 0.08
Reflections collected	33806
Absorption correction	Muti-scan
Data / restraints / parameters	6480/453/643
Goodness-of-fit on F2	1.026
R indices (all data)	R1 = 0.0845 wR2 = 0.2382

Table S5. Representative bond lengths [Å] for [**2**–**Br**].

	**[2–Br]**
Mn-C1	1.803
Mn-N1	2.044
N1-C2	1.343
C2-C3	1.384
C3-C4	1.382
C4-C5	1.386
C5-C6	1.391
C6-C7	1.472
C7-C8	1.390
C8-C9	1.384
C9-C10	1.386
C10-C11	1.388
C11-N2	1.349
Mn-N2	2.090
Mn-C13	1.823
Mn-C14	1.809
C1-O1	1.151
C13-O2	1.147
C14-O3	1.131

Table S6. Crystal data and structure refinement for [**2**–**Br**].

Empirical formula	$C_{16}H_{13}N_3O_4MnBr$
Formula weight	446.13
Temperature	100 K
Wavelength	0.71073
Crystal system	Monoclinic
Space group	P 21/n
Unit cell dimensions	a = 10.5955(4) Å α = 90º<br>b = 10.5011(4) Å β = 94.048º<br>c = 15.0027(6) γ = 90º
Volume	1665.10(11) Å^3
Density (calculated)	1.780 mg/m^3
Absorption coefficient	3.219
F(000)	888.0
Crystal size	0.28 x 0.24 x 0.2
Reflections collected	26780
Absorption correction	Muti-scan
Data / restraints / parameters	3848/0/229
Goodness-of-fit on F2	1.023
R indices (all data)	R1 = 0.0227 wR2 = 0.0515

Table S7. The current density of first and second oxidation wave for complex [**1**–**Br**] in Ar-saturated CH_3CN with 0.1 M TBAP at different scan rate.

**Scan rate (V s^{-1})**	***i*$_{ox1}$ (mA cm^{-2})**	***i*$_{ox2}$ (mA cm^{-2})**	***i*$_{ox1}$/*i*$_{ox2}$**
0.1	0.02	0.11	0.18
0.2	0.05	0.16	0.31
0.4	0.14	0.34	0.41
0.6	0.21	0.28	0.75
0.8	0.31	0.35	0.89
1.0	0.43	0.39	1.10
1.2	0.5	0.42	1.19

Table S8. Catalytic data of selected Mn-based homogeneous catalysts together with the standard CO_2 reduction potentials and electrode equilibrium potentials. All the scan rates are at 100 mV s^{-1}.

**Catalyst**	Solvent	E^o (V)	E_{eq} (V)	η (V) at catalytic current density −1.0 mA cm^{-2}	Refs.
[**1**–**Br**]	CH_3CN + 9% (5.51 M) H_2O	−1.37	−1.17	0.34	This work
[**2**–**Br**]	CH_3CN + 9% (5.51 M) H_2O	−1.37	−1.17	0.76	This work
[**3**–**Br**]	CH_3CN + 9% (5.51 M) H_2O	−1.37	−1.17	0.84	This work
[Mn(bpy-^{t}Bu)$(CO)_3$Br]	CH_3CN + 5% H_2O	−1.43	−1.23	0.61	a)
[Mn(mesbpy)$(CO)_3$(MeCN)](OTf)	CH_3CN + 13% H_2O	−1.31	−1.11	0.81	b)
[Mn(($(MeO)_2$Ph$)_2$bpy)$(CO)_3$(MeCN)](OTf)	CH_3CN +13% H_2O	−1.31	−1.11	---	c)
[Mn(dhbpy)$(CO)_3$Br]	***CH_3CN***				d)
[Mn(ptbpy)$(CO)_3$Br]	CH_3CN + 5% H_2O	−1.43	−1.23	0.92	e)
[Mn(HOPh-bpy)$(CO)_3$Br]	CH_3CN + 5% H_2O	−1.43	−1.22	0.37	f)
[Mn(Me(ImMe)bpy)(bpy)Br](PF_6)	CH_3CN + 19% H_2O	−1.28	−1.08	0.44	g)
[Mn(Me-Im-Py)$(CO)_3$Br]	CH_3CN + 5% H_2O	−1.43	−1.23	---	h)

a) Smieja, J. M.; Sampson, M. D.; Grice, K. A.; Benson, E. E.; Froehlich, J. D.; Kubiak, C. P. *Inorg. Chem.* **2013**, *52*, 2484; b) Sampson, M. D.; Nguyen, A. D.; Grice, K. A.; Moore, C. E.; Rheingold, A. L.; Kubiak, C. P. *J. Am. Chem. Soc.* **2014**, *136*, 5460; c) Ngo, K. T.; McKinnon, M.; Mahanti, B.; Narayanan, R.; Grills, D. C.; Ertem, M. Z.; Rochford, J. *J. Am. Chem. Soc.* **2017**, *139*, 2604; d) Franco, F.; Cometto, C.; Vallana, F. F.; Sordello, F.; Priola, E.; Minero, C.; Nervi, C.; Gobetto, R. *Chem. Commun.* **2014,** *50*, 14670; e) Franco, F.; Cometto, C.; Nencini, L.; Barolo, C.; Sordello, F.; Minero, C.; Fiedler, J.; Robert, M.; Gobetto, R.; Nervi, C. *Chem-Eur. J.* **2017,** *23*, 4782; f) Agarwal, J.; Shaw, T. W.; Schaefer, H. F.; Bocarsly, A. B. *Inorg. Chem.* **2015,** *54*, 5285; g) Sung, S.; Li, X. H.; Wolf, L. M.; Meeder, J. R.; Bhuvanesh, N. S.; Grice, K. A.; Panetier, J. A.; Nippe, M. *J. Am. Chem. Soc.* **2019,** *141*, 6569; h) Agarwal, J.; Shaw, T. W.; Stanton, C. J.; Majetich, G. F.; Bocarsly, A. B.; Schaefer, H. F. *Angew. Chem. Int. Ed.* **2014**, *126*, 5252

Table S9. List of computed v_{CO} bands for selected species (scaling factor = 0.957). Experimental values from FTIR-SEC are shown in paranthesis.

Complex	v_{CO} (cm^{-1})	
	Experimental	DFT
[**1**–**Br**]^0	2025, 1932, 1926	2015, 1939, 1929
[**1**–**MeCN**]$^+$	2048,1965, 1949	2036, 1965, 1952
[**1**]^0		1954, 1863, 1852
[**1**$_2$]^0	1978, 1932, 1865 (br)	1955, 1915, 1864-1880
[**1**]$^-$	1917, 1818(br)	1902, 1834, 1823
[**1-CO$_2$**]$^-$	---	1955, 1871, 1864, 1641
[**1-CO$_2$**]$^-$ with 1 H_2O H-bonded	---	1949, 1872, 1860, 1670
[(**1**-H^+)-CO_2H]$^-$	---	1986, 1900, 1893, 1656
[(**1**-H^+)-CO_2H]$^-$ with 1 H_2O H-bonded	---	1993, 1913, 1898, 1653
[**1**-CO_2H]^0	---	2006, 1924, 1907, 1695
[**1**-CO_2H]^0 with 1 H_2O H-bonded	---	2002, 1922, 1903, 1672
[**1**-CO_2H]$^-$	---	1990, 1901, 1885, 1688
[**1**-CO_2H]$^-$ with 1 H_2O H-bonded	---	1990, 1902, 1885, 1688
[**1**-CO]$^+$	---	2100, 2021, 2007, 1978
[**1**-CO]^0	---	2083, 2001, 1984, 1953
